# Ejectosome of *Pectobacterium* bacteriophage ΦM1

**DOI:** 10.1093/pnasnexus/pgae416

**Published:** 2024-09-19

**Authors:** Alice-Roza Eruera, James Hodgkinson-Bean, Georgia L Rutter, Francesca R Hills, Rosheny Kumaran, Alexander J M Crowe, Nickhil Jadav, Fangfang Chang, Klemens McJarrow-Keller, Fátima Jorge, Jaekyung Hyun, Hyejin Kim, Bumhan Ryu, Mihnea Bostina

**Affiliations:** Department of Microbiology and Immunology, University of Otago, Dunedin 9010, New Zealand; Department of Microbiology and Immunology, University of Otago, Dunedin 9010, New Zealand; Department of Microbiology and Immunology, University of Otago, Dunedin 9010, New Zealand; Department of Microbiology and Immunology, University of Otago, Dunedin 9010, New Zealand; Department of Microbiology and Immunology, University of Otago, Dunedin 9010, New Zealand; Department of Microbiology and Immunology, University of Otago, Dunedin 9010, New Zealand; Department of Microbiology and Immunology, University of Otago, Dunedin 9010, New Zealand; Department of Microbiology and Immunology, University of Otago, Dunedin 9010, New Zealand; Department of Microbiology and Immunology, University of Otago, Dunedin 9010, New Zealand; Otago Micro and Nanoscale Imaging, University of Otago, Dunedin 9001, New Zealand; School of Pharmacy, Sungkyunkwan University (성균관대학교), Suwon 16419, South Korea; Research Solution Center, Institute for Basic Science (기초과학연구원), Daejeon 34126, South Korea; Research Solution Center, Institute for Basic Science (기초과학연구원), Daejeon 34126, South Korea; Department of Microbiology and Immunology, University of Otago, Dunedin 9010, New Zealand

**Keywords:** cryo-electron microscopy, bacteriophage, ejectosome

## Abstract

Podophages that infect gram-negative bacteria, such as *Pectobacterium* pathogen ΦM1, encode tail assemblies too short to extend across the complex gram-negative cell wall. To overcome this, podophages encode a large protein complex (ejectosome) packaged inside the viral capsid and correspondingly ejected during infection to form a transient channel that spans the periplasmic space. Here, we describe the ejectosome of bacteriophage ΦM1 to a resolution of 3.32 Å by single-particle cryo-electron microscopy (cryo-EM). The core consists of tetrameric and octameric ejection proteins which form a ∼1.5-MDa ejectosome that must transition through the ∼30 Å aperture created by the short tail nozzle assembly that acts as the conduit for the passage of DNA during infection. The ejectosome forms several grooves into which coils of genomic DNA are fit before the DNA sharply turns and goes down the tunnel and into the portal. In addition, we reconstructed the icosahedral capsid and hybrid tail apparatus to resolutions between 3.04 and 3.23 Å, and note an uncommon fold adopted by the dimerized decoration proteins which further emphasize the structural diversity of podophages. These reconstructions have allowed the generation of a complete atomic model of the ΦM1, uncovering two distinct decoration proteins and highlighting the exquisite structural diversity of tailed bacteriophages.

**Significance Statement**

This study resolves the cryo-EM structure of bacteriophage ΦM1 infecting the potato pathogen *Pectobacterium atrosepticum*. The capsid features unique decoration proteins on the capsid, exhibiting a fold and arrangements rarely observed in tailed phages. Significantly, we report the preejection state of ΦM1's ejectosome, essential for assembly and DNA injection into the host, showing marked structural differences from the T7 ejectosome in size, structure, and domain composition. Lastly, we offer a detailed method for reconstructing ejectosomes using standard cryo-EM software, aiding future studies of macromolecular structures with isomeric components.

## Introduction

By current estimates, there are ∼10^31^ bacteriophage particles in the biosphere, which equates to 10 times more bacteriophages on the planet than bacterial hosts (1, 2). Tailed bacteriophages of the order *Caudoviricetes* are estimated to have emerged during the early Precambrian period, making bacteriophages not only the most abundant biological agent on the planet, but also among the oldest (2, 3). Through such an extensive evolutionary history, phages have evolved exquisitely diverse and mosaic genomes and are collectively capable of infecting essentially all known bacterial hosts (4, 5). Tailed bacteriophages are constrained to three major morphotypes based on tail morphology; the short noncontractile podophages, the long noncontractile siphophages, and the long contractile myophages. Despite significant conservation in structure, particle assembly, and function amongst these morphotypes, mechanisms of host cell penetration and genomic ejection vary considerably (6).

Podophages belonging to the family *Autographiviridae*, otherwise known as the T7-like supergroup, possess noncontractile tails shorter than the width of the bacterial cell envelope (7–9). Some podophages translocate their genome into the host cytoplasm via a set of internal proteins known to form a protective channel that spans the entire width of the gram-negative cell wall (10–12). Collectively, this internal complex is referred to as the internal core, or the ejectosome. The ejectosome is composed of an assembly of three proteins housed inside the capsid, coaxially arranged as rings mounted on the crown or barrel domain of the phage portal (13, 14). The ejectosome is expelled through the relatively narrow tail channel into the periplasmic space of the cell wall ahead of the genomic DNA. Within the periplasmic space, the proteins rapidly reassemble into a trans-envelope tunnel, subsequently acting as a conduit for the viral DNA to pass through the periplasmic space, preventing degradation by host nucleases or damage by the oxidizing periplasmic environment (8, 15). The T7 tetrameric ejection protein (TEP), gp16, encodes an LTase domain known to locally degrade peptidoglycan during this process, allowing complete penetration of the cell wall (8, 9). After genome delivery into the cytoplasm, the ejectosome rapidly disassembles and the cell wall is mended, preventing premature cell lysis (8, 14, 15).

Despite the recent publication of several high-resolution whole particle podophage structures known to encode ejectosomes (e.g. HRP29, P-SCSP1u, GP4, carin-1), only the *Escherichia coli* podophage T7 ejectosome has been successfully reconstructed (14–18). The reconstruction procedure is technically challenging and the lack of available reference models makes tertiary structure prediction tools like AlphaFold unreliable. Indeed, ejection proteins simply remain as annotated features in the sequenced genomes of many bacteriophages or are occasionally identified as disordered densities in map slices through asymmetric reconstructions (15–18).

In 1995, an investigation into new transducing phages for use as genetic tools in *Pectobacterium atrosepticum* leads to the discovery of bacteriophage ΦM1 which was later shown to generate mutants capable of escaping an abortive infection mechanism in the host induced by a Type III protein–RNA toxin–antitoxin system (19, 20). The receptor remains unknown. Phage ΦM1 is grouped into genus “*Phimunavirus*,” of which members are referred to as “ΦM1-like” (21). Phage ΦM1 encodes a DNA genome ∼44 kb long, organized into 52 putative genes. The host bacterium for ΦM1, *P. atrosepticum*, is a global gram-negative potato pathogen found in both temperate and tropical regions and is known to colonize the intercellular spaces of plant cells and degrade the cell wall, causing blackleg disease and aerial stem rot (22, 23). Recent studies have focused on identifying biocontrol agents against *P. atrosepticum*, including antimicrobial chemicals, zinc/silver nanoparticles, and antagonistic bacterial isolates (24–27). Comparatively, little research has been done on the potential of phage-based biocontrols.

Using cryo-EM, we investigated the whole particle structure of bacteriophage ΦM1, identifying two decorations with relatively uncommon morphology, and a tail fiber/spike assembly with features similar to the recently published structure of Shigella phage HRP29 and phage T7. We describe an odd and unexpected capsid dimerization phenomenon, which we speculate represents an aberrant population of particles that form early in particle maturation. We pay particular focus to the structure of the ΦM1 ejectosome, which shows significant structural divergence relative to bacteriophage T7. We identify and describe a pattern of spooling density around the exterior of the ejectosome machinery, which may represent strands of dsDNA. And finally, we provide a clear cryoSPARC-based workflow for reconstruction of podophage ejectosomes and describe an intrinsic complication in the asymmetric reconstruction of podophages that has likely limited success in previous reconstruction attempts by others.

## Results

### Structural organization of bacteriophage ΦM1

**Fig. 1.**
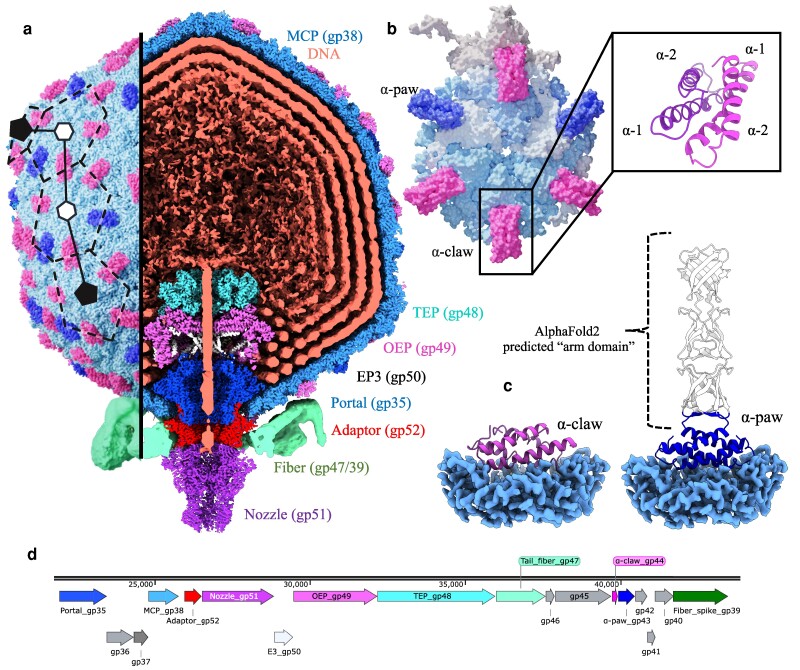
An overview of bacteriophage ΦM1. a) Complete aligned composite reconstruction of the entire bacteriophage ΦM1 particle. b) Asymmetric unit displayed in molecular surface representation. Homodimers of the α-paw protein (dark blue) and the α-claw protein (pink) decoration proteins that bind the 2-fold and quasi-2-fold axis in an alternating pattern dictated by pentamer proximity. MCPs that contact α-claw proteins are colored light blue. MCPs that contact α-paw proteins are colored white. The pentameric MCP is colored cream. Boxed on the right is a depiction of the α-claw motif which is conserved in both decoration proteins. c) In situ depiction of α-claw and α-paw proteins. The unmodeled α-paw arm domain predicted by AlphaFold2 multimer is shown in white. d) Schematic of the ΦM1 structural module (generated in SnapGene V7.0.2).

Here, we report a near-complete atomic model of the bacteriophage ΦM1. While mass spectrometry was not performed on the ΦM1 sample, peptide allocations were possible through fitting and refinement of high-confidence AlphaFold2 models. Bacteriophage ΦM1 encloses the viral DNA within a highly decorated T = 7 icosahedral capsid (Figure 1a, Fig. S12) composed of HK97-like major capsid proteins (MCPs) and two similar, but distinct, accessory proteins (Figure 1a and b). The capsid houses a large ejectosome complex composed of TEPs (, gp48), octameric ejection proteins (OEPs, gp49), and ejection protein 3 (EP3, gp50) arranged axially above the crown of the portal complex (gp35). An adaptor assembly (gp52) interfaces with the portal and six tail fibers (trimeric gp47), each of which appears composed of long helical bundles much like phage T7, as well as adapts trimers of spike protein (gp39) which may be homologous to those in phage P22, another podophage, and 9NA, a siphovirus (Fig. S11) (28). Gp39 was poorly ordered in our reconstructions and was not modeled. However, AlphaFold2 multimer predicts a structure similar to phage HRP29 gp52. The short tail assembly has the closest similarity to bacteriophage HRP29 (16). Summaries of model building validations and missing residues are given in Tables S3–S7.

Two decoration proteins, gp44 and gp43, are individually expressed off the viral genome and bind across MCPs at the 2-fold and quasi-2-fold axes of symmetry via formation of homodimeric α-helical bundles, where each dimerization domain consists of a helix–turn–helix motif (Figure 1c). We refer to these bundle structures as α-claw motifs, as corresponding density bears resemblance to claw clips. The gp44 consists only of the α-claw motif, which we refer to as the α-claw protein. Gp43, however, also encodes a long helical fiber that we term the “forearm” domain. We refer to gp43 as the α-paw protein. While the α-claw is a small protein (∼6 kDa) and is composed primarily of two α-helices, the α-paw is nearly three times the size (∼17 kDa), due to the long C-terminal forearm domain (Figure 1c). Likely due to flexibility, the forearm domain was not well resolved, but can be observed at low contour values (Fig. S13).

Empty capsids reconstructed from the data revealed a number of aberrant capsid-complex products which dimerize at the tail end into a structure similar to previously observed structures in phage T7 tailless mutants (Fig. S1) (29). A low-resolution (∼10 Å) D12 reconstruction of a capsid dimer species showed density for the portal and adaptor complexes of the neck (Figure 1d and e), but no density for the ejectosome, tail fibers or the nozzle. Inspection of the dimer interface between symmetrized adaptor models auto-fit into the map suggested that this oligomerization likely forms due to formation of a putative reciprocal salt bridge network, forming between residues Arg25 and Asp23 of each chain of gp52 (Fig. S1). This hypothesis is tentative given the poor map resolution. Three species of dimers were observed in our data, empty-to-empty, empty-to-full and full-to-full capsids.

### The α-claw and α-paw decoration proteins

**Fig. 2.**
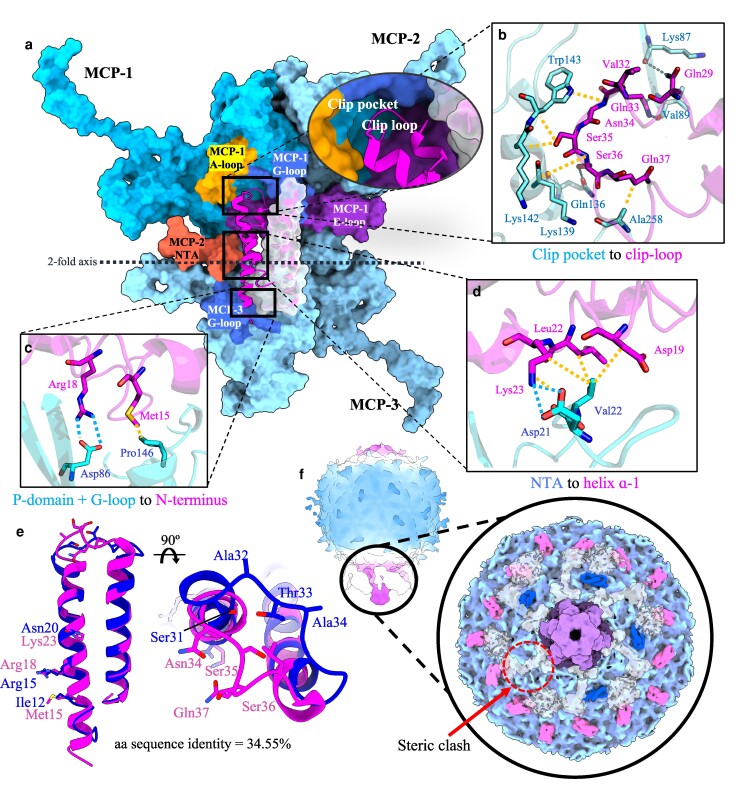
Binding of α-claw motif decoration proteins to the ΦM1 capsid. a) ΦM1 α-claw–capsid surface interface. MCP monomers are labeled MCP-1–3 and are colored different shades of blue to indicate monomer boundaries. A black dotted line shows the 2-fold axis. Major MCP features are colored and labeled according to their identity. Circled is a close up of the conserved clip loop motif binding into the clip pocket, formed by surrounding MCP-1 A-loop (orange), G-loop (royal blue), and E-loop (dark orchid). b) Interactions between the α-claw clip loop and the MCP-1 clip pocket (hydrogen bonds are shown in blue and nonpolar contacts are shown in yellow). c) Interaction between MCP-3 P-domain and G-loop (blue) and the α-claw N-terminus (pink). d) Interaction between the α-claw α-1 helix and the MCP-2 N-terminal arm. e) Overlay of the α-claw and α-paw modeled regions, with key interacting residues highlighted to show conservation/divergence. The α-claw is shown in hot pink and the α-paw is shown in dark blue. f) A low-pass filtered whole particle asymmetric reconstruction (left) and a segmented surface model (right) indicate the distribution of α-claw and α-paw around the unique portal vertex. MCPs are shown in light blue, the nozzle is shown in purple, fiber/spike assemblies are shown in transparent white, the α-claw is shown in hot pink, and the α-paw is shown in dark blue. Density for an α-paw is missing at a probable steric clash site where a fiber lays over the α-paw binding region.

The ΦM1 particle is highly decorated with two accessory proteins, termed the α-claw, and the α-paw. Both proteins bind to 2-fold or quasi-2-fold axes, forming a clip-like structure between opposite and adjacent MCP monomers belonging to adjacent hexamers or pentamers (Figure 2a). The decorations are arranged such that the more common α-claw proteins are bound to all intercapsomeric interfaces, except those immediately flanking the hexamer–pentamer interface. As such, the ratio of α-claw/α-paw is 5:2 per asymmetric unit. The protein fold adopted by these decorations is under-reported, with only one other podophage described with a similar decoration protein fold (16).

Structure alignments of the conserved MCP-binding motif of each protein reveal a pruned RMSD of 0.865 (23 AA) and 8.17 Å across all 46 residues in the motif; additionally, amino acid sequence alignments of the same region showed 34.55% identity. Inspection of the dimerization interface of both α-claw and α-paw homodimers using Proteins Interfaces Surfaces and Assemblies (PISA) analysis showed a strong hydrophobic contribution with only two hydrogen bonds predicted between α-paw monomers (−14.6 ΔG kcal/mol per α-paw dimer, −18.4 ΔG kcal/mol per α-claw dimer) (30). The major point of distinction appears to be in the “clip loop” that connects each protein's helix–turn–helix motif. In both cases, residues in this loop contribute many MCP contacts, and thus, variation in each loop may contribute to the observed variation in binding pattern.

Both proteins make contacts with three MCPs (referred to as MCP-1 to MCP-3 here). The α-claw first contacts MCP-1 via the clip loop, which binds to the clip pocket formed by the A-loop, Spine helix, E-loop, and G-loop of an MCP on one side of the intercapsomeric interface. This is the largest α-claw–MCP interface and appears mostly to involve hydrophobic interactions and hydrogen bonds (Figure 2b). A second interaction occurs with MCP-3 positioned across the intercapsomeric interface and is mediated by a putative salt bridge between Arg18 (α-claw) and Asp86 (MCP) (Figure 2c). A third interaction occurs with the N-terminal arm of MCP-2 forming a putative salt bridge between Asp21 (MCP) and Lys23 (α-claw), in addition to several hydrophobic contacts forming between Val22 (MCP), Lys23, Leu22, and Asp19 (α-claw) (Figure 2d). These monomeric interactions are then approximately reciprocated with the homodimeric partner in an antiparallel fashion. The α-paw protein also makes contacts with three MCPs in the same manner, with some key differences. The α-paw clip loop sits in a shallower position in the clip pocket and is predicted to make weaker interactions (Fig. S2). Additionally, the α-paw encodes an asparagine at position 20, which likely forms hydrogen bonds with Asp21 (MCP), in contrast to the putative salt bridge present at this location in the α-claw protein. Finally, a putative salt bridge is made between MCP Asp86 and Arg18 of the α-paw, or Arg15 of α-claw, respectively. Ultimately, PISA predicts a similar number of salt bridges and H-bonds between MCPs and each decoration protein, although the hydrophobic interface contribution is significantly greater in the α-claw (−1 ΔG kcal/mol per α-paw dimer, −8.6 ΔG kcal/mol per α-claw dimer).

As changes in the local capsid environment have been previously shown to modify the distribution of decoration proteins (31), we inspected how the unique portal vertex affects the distribution of both the α-claw and α-paw via asymmetric reconstructions. As might be expected, α-claw proteins that would normally bind the pentamer–hexamer interface were absent, due to the lack of reciprocating pentameric MCPs. The distribution of α-paw proteins immediately flanking the unique vertex was almost identical, with the exception that a single α-paw dimer was absent at a single interface (Figure 2f). This interface sits directly below one subunit of the tail fiber/spike complex, and we attribute this absence to steric hindrance.

### Organization of ΦM1 ejectosome

**Fig. 3.**
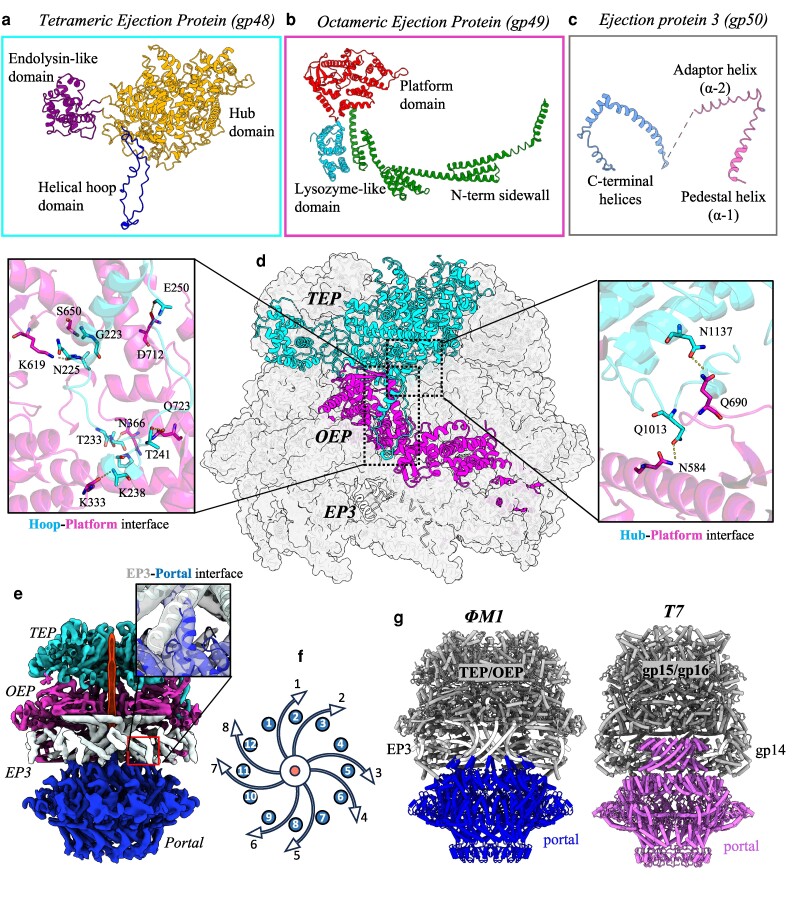
Architecture of ΦM1 ejectosome. Subdomains of a) TEP , b) OEP, and c) EP3 are labeled. Where a domain is sufficiently conserved with the homolog of bacteriophage T7, the naming convention of T7 has been preserved. d) One chain of the TEP, OEP, and EP3 is colored, with symmetry mates of the respective assemblies displayed as silhouettes. Two interfaces of the TEP and OEP are displayed with relevant polar contacts shown between sidechains. e) The consensus map of the ejectosome is displayed on top of the portal assembly with a strand of genomic DNA shown in red within the DNA channel. The model shown is a rigid-body fit of the AlphaFold2 model into a low-resolution 3D class where the ejectosome and portal were aligned (deposited under EMDB code: EMD-43110). f) A schematic of the axial arrangement at the EP3–portal interface. The EP3 α-2 helices (white arrows) adapt to the 12 helices of the portal crown. The central tube of DNA is represented in red. g) The ejectosome of both phages is shown in white, with the portal assembly shown in blue (ΦM1, *left*) and pink (T7, *right*). A barrel-like pedestal motif is formed by the 12 C-terminal alpha-helices of the T7 portal upon which the ejectosome adapts by slotting around the pedestal. In ΦM1, a homologous pedestal motif is formed by the EP3 of the ejectosome itself, but consisting of eight helices instead of 12, and which adapts to the portal by intercalating with reciprocal C-terminal helices of the crown domain. These EP3 helices are ejected during infection, whereas the pedestal of T7 is not.

Similar to that of bacteriophage T7, the ΦM1 ejectosome is encoded as a three-protein mega complex composed of TEP, OEP, and EP3 oligomers arranged coaxially within the phage capsid (Figure 3a–c). The EP3 octamer adapts the locally 8-fold symmetric interface of the ejectosome to the 12-fold symmetric crown of the portal by intercalating the EP3 helix α-2 between the crown domains of adjacent portal monomers, forming contacts with the C-terminal portal helix (Figure 3e and f). EP3 intercalates in a pattern in which two EP3 α-2 helices intercalate in the gaps between two adjacent portals, followed by an unoccupied gap. This results in four unoccupied portal crown domain gaps, and eight filled with the α-2 helices of the EP3 octamer (Figure 3g).

The ΦM1 ejectosome differs significantly from that of T7 in structure, domain composition of the core proteins, interface interactions, and potential functions (Fig. S3). Through a combination of BLASTP and DALI homology searches, putative enzymatic domains were identified in the ejectosome, specifically a lysozyme-like domain within the OEP (Gly718–Ser903) and an endolysin/exotransglycosidase-like domain within the TEP (Asp268–Asp528) (32, 33). Viral lysozymes and exotransglycosidases can digest peptidoglycan, suggesting that these domains may degrade local peptidoglycan surrounding the periplasmic channel once ejected from the phage particle during infection (34).

During our initial ejectosome reconstructions, portal, EP3, and OEP monomers appeared resolved, while TEP monomers were highly disordered. Through symmetry expansion and 3D classification, we were able to identify two 3D classes with TEP density; in each class, the EP3 and OEP were rotated 45° relative to the other class. A single class was chosen and was further refined with C4 symmetry imposed; however, resulting reconstructions did not feature density corresponding to the portal. From this, we deduce that the ejectosome exists in multiple discrete particle species, in which portals cannot be aligned (Fig. S4a and b). A similar phenomenon was previously described in which the portal of podophage SF6 exists in two discrete populations, separated by a 6° rotation about the *Z* axis (35). In our 3D classifications, we also identified two populations of particles in which ejectosome structures were aligned, but portal density was also rotated by ∼6° about the *Z* axis. This suggests that the ΦM1 portal may also exist as two discrete particle populations (Fig. S4c).

### Density spooling around the ejectosome

**Fig. 4.**
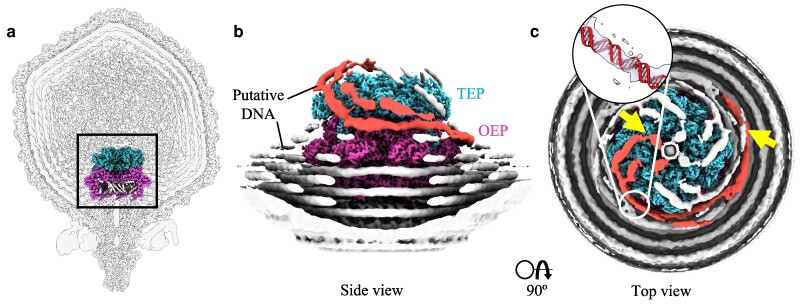
Putative DNA spooling around the ΦM1 ejectosome. a) A phage slice from the asymmetric reconstruction is overlayed with the ejectosome map, showing the relative location of the core proteins within the phage capsid. b) A cropped view of the ejectosome (*TEP* cyan, *OEP* pink, EP3 not shown) from a C4 reconstruction at a 0.064 contour level. Density is shown in white with two spooling strands, assumed to be rotational isomers of the same strand, colored in red. c) The same view is seen showing left-handed spooling toward the central DNA channel within the ejectosome. A piece of dsDNA was produced in Pymol v2.5.5 using the fnab command and placed into a strand (indicated) in UCSF ChimeraX v1.3 to assess the potential fit of dsDNA (map contour level 0.0653). The yellow arrows indicate the starting and ending position of a strand which was measured in UCSF ChimeraX to ∼290–320 Å.

During C4 reconstruction of the ejectosome, disordered density was observed spiraling the outer surface of the ejectosome within 10–15 Å of the OEP and TEP (Figure 4b and c). By applying a strong low-pass filter, we were able to connect these long densities to the density rings that surround the ejectosome and portal, often observed in asymmetric podophage structures and widely accepted as density corresponding to viral DNA (16). In each section of the C4 map, two strands of density follow two alternative routes around the ejectosome, before entering at equivalent positions rotated 90° relative to one another. The density spools upwards in a left-handed manner around the ejectosome toward the capsid center, before making a sharp arch bending downward to join a tube of density that extends into the tail complex, which is presumed to represent the “last-in” stretch of dsDNA. For each pair of strands, which may represent multiple local conformations of entry, one strand coils more tightly around the side of the ejectosome running along a groove formed by the lysozyme-like domains of the OEP and one strand arches over the hub of the TEP. Several attempts were made through focused 3D classification to isolate a single strand of density spooling the ejectosome which could be attributed to genomic DNA spooling around the ejectosome. However, in all attempts, the strands still showed C4 symmetry. Although these strands may represent dsDNA spooling the ejectosome, given they are continuous with the portal-adjacent DNA strand, they cannot be ruled out as reconstruction artifacts. A single spool of putative DNA was measured from the point the strand separates from the lowest DNA ring to the top of the ejectosome (Figure 4) and was roughly 290–320 Å in length, which would correspond to ∼85–94 bp of genetic information (1 bp = 3.4 Å) (36).

## Discussion

ΦM1 can be thought of as an arrangement of protein oligomeric rings represented by C4, C5, C6, C8, and C12 symmetries. Such a wide array of symmetries creates several symmetry breaking interfaces which complicate the process of reconstructions with imposed symmetry. However, in this study, we discovered these boundaries also introduced difficulties in asymmetric reconstruction. The C12 portal complex that sits in the locally C5 MCP environment results in a symmetrically incompatible interface that is best characterized by C1 symmetry. However, if we consider the case of the portal/EP3 interface, and the OEP/TEP interface, multiple symmetrical positions become possible, complicating reconstruction procedures. Our workflow (symmetry expansion followed by 3D classification) permits the refinement of particle orientations such that all ejectosome components are aligned. However, the consequence of refining the alignment of ejectosome components was loss of the EP3/portal interface, and total loss of portal density. Further, during whole particle asymmetric reconstruction, capsids, nozzles, fibers, and portals appear aligned, while the ejectosome showed visible but highly disordered density. Therefore, the TEP tetramer positions itself on top of the OEP octameric interface in two discrete rotameric populations that cannot be aligned during asymmetric reconstruction (Fig. S4a and b).

Analysis of other recent whole particle asymmetric podophage reconstructions (e.g. HRP29, P-SCSP1u, GP4, carin-1) suggests a similar phenomenon in which the ejectosome is visible in real space slices but was not reconstructed to high resolution (14–17). We hypothesize this might be caused by the improper sorting of whole particle rotamer species, resulting in misaligned internal proteins. By focusing only on the ejectosome, our reconstruction workflow allows ejectosome orientations to be refined such that ejectosome components are *correctly* aligned to each other, resulting in a high-resolution reconstruction with the lowest order of symmetry imposed (C4). We acknowledge this was previously observed in low-resolution bacteriophage T7 maps (37). Exploration of the extent to which rotamer species affect asymmetric reconstruction of bacteriophages represents a future area of investigation.

Reconstruction of capsid dimers to low resolution reveals a probable interface forming between opposite and opposed adaptor proteins. It is unclear at exactly what stage of maturation these dimers may have formed given the range of morphologies observed. It is possible that empty–empty dimers form due to tail breakage postgenome ejection, resulting in the formation of a dimerization interface. We find this unlikely given we also observe empty–full and full–full dimers as well, leading one to expect that dimerization likely occurs shortly after (or before) genomic packaging. It is possible that empty–empty dimers form due to premature oligomerization of adaptors to the portal complex, preventing terminase association and subsequent DNA packaging. Alternatively, these capsids may represent an aberrant particle population unable to interface with the terminase motor to begin with. While these dimers have been observed previously in T7 tailless mutants, it is unknown whether this analogous structure would occur naturally in native T7 particle populations, or as a by-product of the mutations that prevent tail attachment (29). The latter is a possibility given the known hydrophobic patch formed on the interface surface of T7 adaptor complexes, and the lack of conservation of a positively charged residue at the position analogous to ΦM1 Arg25, preventing formation of the putative salt bridge network described here (29, 38). The physiological relevance of capsid dimers is unclear, but we suspect that these dimers represent an aberrant noninfectious product of capsid maturation.

The α-claw and α-paw decoration proteins, although similar in the helix–turn–helix domain, may serve different functions during infection by virtue of their structures. Both decorations likely stabilize the capsid as they bind across MCPs, which is a common function for accessory proteins in large capsid viruses which can reach up to 60 atmospheric units of internal pressure once packaged (39). Given that the α-claw appears to form a stronger interface with MCPs and does not encode any additional fiber, we hypothesize that this protein is required to maintain capsid stability. In contrast, the α-paw likely contributes to stability to a lesser extent but may mediate additional functions via the fibrous α-paw forearm domain, such as host recognition and adhesion. Similar functions have been observed in the decoration proteins of other DNA phages, e.g. phage N4 gp17, phi29 gp8.5, bacteriophage T5 pb10, and T4 HOC (39–42). It should be noted that unlike the α-paw, which binds to quasi-2-fold axes, these decorations bind to hexamer centers and have entirely unrelated structures. Inspection of the α-paw forearm domain AlphaFold2 prediction in electrostatic and hydrophobicity representation reveals a relatively hydrophilic and uncharged surface, with no clear interface surface; however, this might be explained by errors in prediction, or environmentally dependent changes in structure (e.g. pH, ionic composition). Analysis of the forearm prediction by DALI, BLASTP, and PredictProtein provided no further insights into putative function (43). The α-claw and α-paw proteins exhibit an alternating pattern across the capsid. In most cases, extended fiber-like domains are found on hexamer-centric decorations (bacteriophage N4 gp17, phi29 gp8.5, T5 pb10, and T4 HOC), rather than decorations adhered to 2-fold axes (39). Despite the fact that fibrous decorations that bind to these axes could be arrayed at higher copy numbers, the limited adherence of α-paws to the ΦM1 capsid results in only a single complete α-paw dimer per asymmetric unit, limiting the stoichiometry of fiber-like protrusions to that of hexamer-centric decorations. The significance of this limited array of α-paws cannot be commented on without further knowledge of α-paw function. Future work will focus on a more thorough investigation of α-paw and α-claw relative functions via deletion/knockout experiments, to shed further light on potential advantages of this decoration arrangement.

Previous authors have hypothesized that the co-axial spooling of podophage genomes is likely influenced by internal structures such as the portal, or the ejectosome, sighting particularly that DNA appears more ordered in close proximity to the portal/core (44–46). We found this to be true for all co-axial DNA rings observed. However, here we also show direct evidence of how the ejectosome alters the path of DNA density. Strands of density we ascribe to dsDNA were observed in our low-pass filtered maps in two positions before turning sharply to enter the central channel and extend toward the tail. Given the densities' diameter and continuity with dsDNA ring densities, we are confident that these densities correspond to dsDNA, and not an alternate packaged material, e.g. additional hypothetical protein products or unmodeled residues. Density is always observed to enter the ejectosome at approximately the same position, suggesting that some force is constraining the final packed dsDNA in a highly ordered manner. From this, we speculate the ejectosome itself directly or indirectly curates the structural organization of at least the last-in packaged DNA. In the direct case, disordered ejectosome loops may anchor last-in DNA along a specific path; such disordered loops can be observed in the OEP between residues L771-E783, and in the TEP endolysin-like domain between residues R280-S288, S321-A356, and V427-D446. Each of these loops contains positively charged residues and is proximal to the coil path of dsDNA. In the indirect case, the pattern of DNA coiling around the ejectosome may be due to repulsive electrostatic interactions forcing DNA to occupy the space most frequently unoccupied, in this case, the groove formed by the endolysin and lysozyme-like domains of TEP and OEP, respectively. This may explain why such an interaction was not so easily observed in phage T7, which lacks these grooves. Although a nonsymmetrical DNA strand could not be obtained, this may be due to insufficient particle distribution and/or flexibility of the DNA associating with the ejectosome, which reduces the efficacy of 3D classification. Whole particle asymmetric reconstructions that utilize a larger dataset may shed some further light on this phenomenon. In any case, these observations clearly demonstrate that genomic organization ΦM1 is extremely ordered, a fact that is likely required for the successful expulsion of not only the highly pressurized genome, but of the ejectosome itself.

Comparison with the ejectosome of T7 reveals a number of important differences. First, the adaption mechanism employed by the EP3 homolog of T7 differs from that of ΦM1. The T7 ejectosome adapts by slipping around a pedestal-like barrel created by the portal crown, whereas the ΦM1 ejectosome adapts on *top* of the portal through intercalating helices which form an analogous barrel. Indeed, a pedestal-like motif exists at the interface between the ejectosome and the portal of both phages, but in T7, this motif consists of 12 C-terminal alpha-helices of the portal, and in ΦM1, is formed by eight helices of the EP3, which are presumably ejected during the expulsion of the ejectosome. It is interesting to note that the EP3 of the ejectosome is able to mimic this structural feature, while still maintaining functionality as an ejectosome protein. Second, the protein domain arrangement of the TEP and OEP proteins differs significantly (Fig. S14). In T7, the TEP consists of an N-terminal LTase followed by the hub domain. However, in ΦM1, the helical hoop and endolysin-like domain are inserted into the hub domain. This significantly alters the interface contacts made between TEP and OEP monomers (Fig. S14c). Further, the ΦM1 OEP has an additional lysozyme-like domain which T7 lacks. The catalytic domains likely serve both phages to degrade peptidoglycan in the cell wall after the ejectosome has been expelled into the host. These differences highlight the evolutionary divergence between the two systems, which may be attributed to host-specific adaptations (T7 in *Escherichia*, ΦM1 in *Pectobacterium*). It is interesting to speculate if the alpha-helical nature of both ejectosomes better permits the dissociation of the complex to allow for partial unfolding and passage down the phage tail, as proposed previously (8, 11).

## Conclusion

We describe an intrinsic complication in the asymmetric reconstruction of podophages that has likely limited success in previous attempts to reconstruct the ejectosome. The ejectosome proteins of ΦM1, despite clear structural homology, appear highly divergent in both structure and sequence when compared with the T7 ejectosome. In this study, we clearly describe a cryoSPARC-compatible reconstruction process to produce structures of the ejectosome of ΦM1 by single-particle cryo-EM. We describe density spooling around the ejectosome in a left-handed manner before exiting the capsid and extending into the tail, which may be genomic dsDNA. In addition, two decorations bind the capsid across adjacent MCPs via a rarely observed structural motif, potentially stabilizing the capsid and engaging in host adhesion. Further, capsids or capsid-like products appear to dimerize at the connector interface, which may happen during an early stage in particle maturation or perhaps after genome ejection. The methods described in this study will support the efforts in the reconstruction of symmetric and asymmetric elements of phages.

## Materials and methods

### Culturing of *P. atrosepticum* and phage purification

For lysate preparation, 6 mL of *P. atrosepticum* overnight culture was added to 200 mL low-NaCl LB (10 g/mL tryptone, 5 g/mL yeast extract, 5 g/mL NaCl), and was incubated for 1.5 hours at 25°C. To the culture, 400 mL of high-titer ΦM1 sample was added and incubated at 25°C for 17 hours. Crude lysates were centrifuged at 10,000 ✕ g for 20 minutes (Thermo Fisher Lynx 6000, Fiberlite F12-6 × 500 LEX rotor) at 20°C and decanted into an autoclaved Schott bottle. Supernatant containing phage was filtered using a 0.22-μm filter. Sample was loaded into polypropylene thin-walled 38.5-mL ultracentrifuge tubes (Beckman Coulter, ref. no. 326823) underlaid with 3 mL of 20% w/v sucrose and filled with phage buffer (10 mM Tris–HCl pH 7.4, 10 mM MgSO_4_, and 0.01% w/v gelatin). Tubes were loaded into SW32 swing buckets. The sucrose cushion was spun at 50,000 ✕ g for 1.5 hours at 20°C (Sorvall™ WX + Thermo Fisher, Beckman SW 32 Ti rotor). Sample supernatant was decanted, and residual buffer was tapped out of tubes. All six pellets were resuspended in 500 mL of phage buffer overnight at 4°C. Pellets were pooled and diluted with phage buffer to a total volume of 22.5 mL. Gradients were prepared using 4 ✕ 1.5 mL of CsCl at densities of 1.33, 1.45, 1.6, and 1.7 g/cm^3^ with the lightest density at the top and the heaviest density at the bottom, prepared in thin-walled 17-mL polypropylene ultracentrifuge tubes (Beckman Coulter, ref. no. 337986). To both gradients, 11 mL of sample was loaded. Gradients were transferred into Beckman SW32.1 swing buckets and centrifuged at 100,000 ✕ g for 4 hours at 20°C. Thick bands were visible postcentrifugation at the interface between 1.45 and 1.6 g/cm^3^. Bands were carefully harvested followed by concentration and buffer exchanging into phage buffer using Amicon 100 kDa molecular weight cutoff 500 mL concentrators centrifuged at 14,000 ✕ g at room temperature. Samples were stored at 4°C for later use.

### Negative-stain electron microscopy

Specimens were assessed for sample quality and purity by negative-stain EM on a JEOL 1400 FLASH transmission electron microscope (TEM). Sample was loaded onto C-flat 300-nm mesh carbon coated grids followed by plasma discharge with negative charge using the GloQube discharge system (Quorum Technologies) for 30 seconds at 15 mA. Next, 5 μL of sample was applied to the carbon film and incubated for 60 seconds followed by negative staining with 5 µL of 1% phosphotungstic acid. Grids were air-dried and imaged in the JEOL 1400 FLASH TEM operating at 120 kV, for sample quality, defined by intact particle appearance, good particle distribution, and lack of obvious specimen contaminants. Of note, we observed almost all phage particles to have ejected their genomic material in the negatively stained images. This was in stark contrast to later cryo-EM imaging of the same phage sample which showed primarily full particles.

### Cryo-EM sample preparation and data collection

Quantafoil R1.2/1.3 300 mesh grids were negatively glow discharged for 30 seconds at 45 mA, and 3 µL of purified M1 phage was applied. Grids were plunge frozen using a FEI Mark IV Vitrobot (Thermo Fisher) with a blot force of 0, a blot time of 3, a 100% humidity, a temperature of 4°C, and a wait time of 30 seconds. Data were collected using EPU software on two FEI Titan Krios G4 microscopes (Thermo Fisher), both operating at 300 kV with a K3 BioQuantum Gatan camera system. Exposures were collected for 12 seconds with an accumulated electron dose of 54 e/Å^2^, fractionated into 50 frames. Exposures were collected with a range of defoci from −0.8 to −2.4 µm. The calibrated pixel size was 1.36 Å for one dataset and 1.40 Å for the other, and the lower pixel size dataset was scaled for data merging purposes adapting the methods of Wilkinson et al. (47). A figure briefly detailing the scaling and merging method is shown in Fig. S5.

### Data merging and processing

Data were collected across multiple sites (Institute of Basic Science [IBS], Daejeon, and Sungkyunkwan University [SKKU], Suwon), each reporting slightly different pixel sizes (SKKU: nominal 1.40 Å, IBS: calibrated 1.36 Å). For data merging, the IBS dataset was rescaled to the larger pixel size of SKKU data to facilitate data merging for downstream processing (47). The reader is directed to Figs. S5 andS6 for a workflow diagram of the process. Both datasets were aligned, motion-corrected, and dose-weighted using the Patch-Based Motion Correction function in cryoSPARC v4.2.1 (48). Icosahedral capsid reconstructions were then produced independently from a subset of both datasets using standard reconstruction methods outlined below and were imported into UCSF ChimeraX for real space cross-correlation analysis between the two reconstructions using the fitMap tool (49). Peak cross-correlation between the maps was observed at IBS: 1.394 Å, SKKU 1.400 Å. For the IBS dataset, micrographs were rescaled using this output to a final pixel size of 1.400 Å using Relion_Image_Handler (50). The micrographs were then imported into cryoSPARC and merged with the SKKU dataset. Micrograph stacks were then submitted to independent patch CTF correction. The combined micrograph stack contained 4,429 micrographs. All downstream data processing was performed in version 4.2.1 of cryoSPARC. Briefly, global contrast transfer functions (CTF) were fitted using the patch-based CTF correction function and particles were picked using automated blob picking and subjected to iterative 2D classing until classes with clear secondary features were produced. Due to the modular nature of bacteriophages, downstream reconstruction workflow deviated significantly for different parts of the phage to best utilize available symmetry information.

### Capsid reconstruction

For the capsid, multiple rounds of 2D classification yielded ∼18,000 full capsid particles. Good particles were submitted to ab initio reconstruction without a symmetry group applied, and the best class was selected for further refinement using the homogeneous refinement job with I2 symmetry imposed, followed by several rounds of nonuniform refinement in I2 symmetry with per particle defocus and negative Ewald sphere correction applied. Final DNA-full capsid maps were produced at a global resolution of 3.14 Å.

### Tail reconstruction

For tail reconstructions, tail particles were identified by the following workflow. An early, strongly Fourier-cropped (4✕) capsid reconstruction with imposed icosahedral symmetry was used to identify vertex coordinates spaced ∼50 Å from the vertex surface using an in-house python script and UCSF ChimeraX. We note that distance from the vertex surface and box size appears to be important and sensitive variables in the success of this workflow and iterative optimization is strongly recommended. Particles aligned to this capsid reconstruction were symmetry expanded using I2 symmetry, and particle box centers were re-centered to the new vertex coordinates using a Volume Alignment Tools job in cryoSPARC. Particles were then extracted with a smaller Fourier-cropped box size (360 pix → 180 pix), followed by removal of duplicate images using a 40 Å distance threshold. The particle stack (now ∼12 ✕ original size) was submitted to 3D classification without alignment. Vertex particles containing tails were pooled into one class approximately equal to the original stack size, which was then used as the unique vertex particle stack in downstream tail, fiber, and internal protein reconstructions. Tail reconstruction was performed first by homogenous refinement with C6 symmetry imposed, followed by local CTF correction and nonuniform refinement. A final resolution of 3.23 Å was achieved for the full C6 symmetrized reconstruction. To resolve the C12 portal and adapter portions of the tail, particle centers were shifted 163 Å in Z, followed by reconstruction without alignment to produce an initial model with C12 components centered. This model was then refined with C12 symmetry using the local refinement job with a supplied focus mask, followed by re-estimation of local CTF and nonuniform refinement. The final full C12 symmetrized tail reconstruction resolution was 2.98 Å.

### Internal protein (ejectosome) reconstructions

A full reconstruction protocol is available in Supplementary Method 1. Briefly, C12 tail coordinates described in the tail reconstruction section were re-centered to achieve a 165 Å shift in Z toward the capsid center, followed by re-extraction and reconstruction without alignments and a C4 local refinement with a large nonspecific focus mask. Next, C12 symmetry expansion was performed followed by 3D classification to identify 3D classes with correctly aligned ejectosomes. Particles from one representative class were used for further local refinements in C1 symmetry. Through this method, C8 components corresponding to OEP and EP3 were recovered; however, TEP appeared to be absent in reconstructions. To resolve this, symmetry expansion using C8 symmetry was performed followed by 3D classification, which yielded a complete ejectosome class for final local refinements. Duplicate particles were removed to effectively reverse symmetry expansion, resulting in a particle stack approximately equal to the initial stack size suitable for C4 reconstructions. This particle set was submitted to local CTF estimation and local refinement using a tighter focus mask as this method appears to yield the best results for proteins embedded in somewhat disordered DNA such as the portal and ejectosome. The final ejectosome reconstruction yielded a resolution of 3.32 Å. Note that the EP3 was modeled into the C8 ejectosome map but deposited in the C4 map.

### Model building and refinement

Most preliminary models were produced in Alphafold2 using Google Colab (51) and fit into consensus maps, either segmented or whole, using UCSF ChimeraX (49) followed by manual refinement in COOT and ISOLDE (52, 53). In the case of the collar fiber trimer proteins, AlphaFold multimer was used to produce the initial models using the Cosmic^2^ server (54). For the TEP, which was particularly challenging given the uniqueness of the structure and many disordered loops within the map, an initial model was generated in AlphaFold2 but required additional efforts as only a small domain fit in the segmented map. The homologous protein to TEP from bacteriophage T7, which possesses a small conserved domain, was fit into the TEP map as best as practicable, and the two models were then merged via Swiss Model (https://swissmodel.expasy.org) to create a better fitting model for the density (55). Most of this model then refined into the map as usual, although the first 300 N-terminal residues required modeling individually. Geometries were corrected in COOT and ISOLDE, and models were symmetrized to fit into the final consensus map. Model validation was performed by Molprobity score (https://molprobity.biochem.duke.edu/) (56).

## Supplementary Material

pgae416_Supplementary_Data

## Data Availability

The maps and models for the ΦM1 phage have been deposited in the Protein Data Bank (PDB) and Electron Microscopy Data Bank (EMDB), https://www.ebi.ac.uk/emdb/with accession codes 8VB0 and EMD-43109 for the asymmetric unit, 8VB4 and EMD-43112 for the portal-adaptor assembly, 8VB2 and EMD-43110 for the ejectosome, 8VBX and EMD-43127 for the spike-nozzle assembly, respectively. The C8 ejectosome map and asymmetric whole particle map was deposited to the EMDB under EMD-43111 and EMD-43132, respectively.

## Preprints

This manuscript was posted on a preprint server: https://doi.org/10.1101/2024.02.19.577657.

## References

[1] Hatfull GF , Hendrix RW. 2011. Bacteriophages and their genomes. Curr Opin Virol. 1:298–303. 22034588 10.1016/j.coviro.2011.06.009 PMC3199584

[2] Hatfull GF . 2008. Bacteriophage genomics. Curr Opin Microbiol. 11:447–453. 18824125 10.1016/j.mib.2008.09.004 PMC2706577

[3] Ackermann HW . 1998. Tailed bacteriophages: the order caudovirales. Adv Virus Res. 51:135–201. 9891587 10.1016/S0065-3527(08)60785-X PMC7173057

[4] Álvarez-Espejo DM , Rivera D, Moreno-Switt AI. 2024. Bacteriophage-Host interactions and coevolution. Methods Mol Biol. 2738:231–243. 37966603 10.1007/978-1-0716-3549-0_15

[5] Ely B , Lenski J, Mohammadi T. 2024. Structural and genomic diversity of bacteriophages. Methods Mol Biol. 2738:3–16. 37966589 10.1007/978-1-0716-3549-0_1

[6] Dion MB , Oechslin F, Moineau S. 2020. Phage diversity, genomics and phylogeny. Nat Rev Microbiol. 18:125–138. 32015529 10.1038/s41579-019-0311-5

[7] Leptihn S , Gottschalk J, Kuhn A. 2016. T7 ejectosome assembly: a story unfolds. Bacteriophage. 6:e1128513. 27144087 10.1080/21597081.2015.1128513 PMC4836469

[8] Swanson NA , Hou CD, Cingolani G. 2022. Viral ejection proteins: mosaically conserved, conformational gymnasts. Microorganisms. 10:504. 35336080 10.3390/microorganisms10030504 PMC8954989

[9] Casjens SR , Molineux IJ. 2012. Short noncontractile tail machines: adsorption and DNA delivery by podoviruses. Adv Exp Med Biol. 726:143–179. 22297513 10.1007/978-1-4614-0980-9_7

[10] Molineux IJ , Panja D. 2013. Popping the cork: mechanisms of phage genome ejection. Nat Rev Microbiol. 11:194–204. 23385786 10.1038/nrmicro2988

[11] Molineux IJ . 2001. No syringes please, ejection of phage T7 DNA from the virion is enzyme driven. Mol Microbiol. 40:1–8. 11298271 10.1046/j.1365-2958.2001.02357.x

[12] Hu B , Margolin W, Molineux IJ, Liu J. 2013. The bacteriophage t7 virion undergoes extensive structural remodeling during infection. Science (1979). 339:576–579. 10.1126/science.1231887 PMC3873743 23306440

[13] Agirrezabala X , et al 2005. Maturation of phage T7 involves structural modification of both shell and inner core components. EMBO J. 24:3820–3829. 16211007 10.1038/sj.emboj.7600840 PMC1276722

[14] Chen W , et al 2021. Structural changes in bacteriophage T7 upon receptor-induced genome ejection. Proc Natl Acad Sci U S A. 118:e2102003118. 34504014 10.1073/pnas.2102003118 PMC8449382

[15] Swanson NA , et al 2021. Cryo-EM structure of the periplasmic tunnel of T7 DNA-ejectosome at 2.7 Å resolution. Mol Cell. 81:3145–3159.e7. 34214465 10.1016/j.molcel.2021.06.001 PMC8349896

[16] Subramanian S , Bergland Drarvik SM, Tinney KR, Parent KN. 2023. Cryo-EM structure of a Shigella podophage reveals a hybrid tail and novel decoration proteins. Structure. 32:24–34.e4. 37909043 10.1016/j.str.2023.10.007 PMC10842012

[17] d'Acapito A , et al 2023. Structural study of the Cobetia marina bacteriophage 1 (Carin-1) by Cryo-EM. J Virol. 97:e0024823. 36943070 10.1128/jvi.00248-23 PMC10134823

[18] Cai L , et al 2023. Cryo-EM structure of cyanophage P-SCSP1u offers insights into DNA gating and evolution of T7-like viruses. Nat Commun. 14:6438. 37833330 10.1038/s41467-023-42258-7 PMC10575957

[19] Blower TR , et al 2017. Evolution of Pectobacterium bacteriophage ΦM1 to Escape two bifunctional type III toxin-antitoxin and abortive infection systems through mutations in a single viral gene. Appl Environ Microbiol. 83:e03229-03216. 28159786 10.1128/AEM.03229-16 PMC5377504

[20] Watson BNJ , et al 2019. Different genetic and morphological outcomes for phages targeted by single or multiple CRISPR-Cas spacers. Philos Trans R Soc Lond B Biol Sci. 374:20180090. 30905290 10.1098/rstb.2018.0090 PMC6452268

[21] Buttimer C , et al 2018. Pectobacterium atrosepticum phage vB_PatP_CB5: a member of the proposed genus ‘Phimunavirus’. Viruses. 10:394. 30050020 10.3390/v10080394 PMC6115819

[22] Monteagudo-Cascales E , et al 2023. Study of NIT domain-containing chemoreceptors from two global phytopathogens and identification of NIT domains in eukaryotes. Mol Microbiol. 119:739–751. 37186477 10.1111/mmi.15069

[23] Toth IK . 2022. Microbe profile: Pectobacterium atrosepticum: an enemy at the door. Microbiology (Reading). 168:1–3. 10.1099/mic.0.001221 35917166

[24] Hamdy E , et al 2023. Zinc oxide nanoparticles biosynthesized by Eriobotrya japonica leaf extract: characterization, insecticidal and antibacterial properties. Plants (Basel). 12:2826. 37570980 10.3390/plants12152826 PMC10421472

[25] Elkobrosy D , et al 2023. Nematocidal and bactericidal activities of green synthesized silver nanoparticles mediated by Ficus sycomorus leaf extract. Life (Basel). 13:1083 37240728 10.3390/life13051083 PMC10224088

[26] Cigna J , et al 2023. Efficacy of soft-rot disease biocontrol agents in the inhibition of production field pathogen isolates. Microorganisms. 11:372. 36838337 10.3390/microorganisms11020372 PMC9961933

[27] Kang JE , Hwang S, Yoo N, Kim BS, Chung EH. 2022. A resveratrol oligomer, hopeaphenol suppresses virulence activity of Pectobacterium atrosepticum via the modulation of the master regulator, FlhDC. Front Microbiol. 13:999522. 36386642 10.3389/fmicb.2022.999522 PMC9650432

[28] Andres D , et al 2012. Tail morphology controls DNA release in two Salmonella phages with one lipopolysaccharide receptor recognition system. Mol Microbiol. 83:1244–1253. 22364412 10.1111/j.1365-2958.2012.08006.x

[29] Cerritelli ME , et al 1997. Encapsidated conformation of bacteriophage T7 DNA. Cell. 91:271–280. 9346244 10.1016/s0092-8674(00)80409-2

[30] Krissinel E , Henrick K. 2007. Inference of macromolecular assemblies from crystalline state. J Mol Biol. 372:774–797. 17681537 10.1016/j.jmb.2007.05.022

[31] Bayfield OW , et al 2023. Structural atlas of a human gut crassvirus. Nature. 617:409–416. 37138077 10.1038/s41586-023-06019-2 PMC10172136

[32] Holm L , Sander C. 1995. Dali: a network tool for protein structure comparison. Trends Biochem Sci. 20:478–480. 8578593 10.1016/s0968-0004(00)89105-7

[33] Altschul SF , Gish W, Miller W, Myers EW, Lipman DJ. 1990. Basic local alignment search tool. J Mol Biol. 215:403–410. 2231712 10.1016/S0022-2836(05)80360-2

[34] Evrard C , Fastrez J, Soumillion P. 1999. Histidine modification and mutagenesis point to the involvement of a large conformational change in the mechanism of action of phage lambda lysozyme. FEBS Lett. 460:442–446. 10556513 10.1016/s0014-5793(99)01395-2

[35] Li F , et al 2022. High-resolution cryo-EM structure of the Shigella virus Sf6 genome delivery tail machine. Sci Adv. 8:eadc9641 . 36475795 10.1126/sciadv.adc9641 PMC9728967

[36] Mandelkern M , Elias JG, Eden D, Crothers DM. 1981. The dimensions of DNA in solution. J Mol Biol. 152:153–161. 7338906 10.1016/0022-2836(81)90099-1

[37] Guo F , et al 2013. Visualization of uncorrelated, tandem symmetry mismatches in the internal genome packaging apparatus of bacteriophage T7. Proc Natl Acad Sci U S A. 110:6811–6816. 23580619 10.1073/pnas.1215563110 PMC3637776

[38] Cerritelli ME , et al 2003. A second symmetry mismatch at the portal vertex of bacteriophage T7: 8-fold symmetry in the procapsid core. J Mol Biol. 327:1–6. 12614603 10.1016/s0022-2836(03)00117-7

[39] Dedeo CL , Teschke CM, Alexandrescu AT. 2020. Keeping it together: structures, functions, and applications of viral decoration proteins. Viruses. 12:1163. 33066635 10.3390/v12101163 PMC7602432

[40] Fokine A , et al 2023. Structure and function of hoc-A novel environment sensing device encoded by T4 and other bacteriophages. Viruses. 15:1517. 37515203 10.3390/v15071517 PMC10385173

[41] Choi KH , et al 2008. Insight into DNA and protein transport in double-stranded DNA viruses: the structure of bacteriophage N4. J Mol Biol. 378:726–736. 18374942 10.1016/j.jmb.2008.02.059 PMC2396777

[42] Xiang Y , Rossmann MG. 2011. Structure of bacteriophage phi29 head fibers has a supercoiled triple repeating helix-turn-helix motif. Proc Natl Acad Sci U S A. 108:4806–4810. 21383126 10.1073/pnas.1018097108 PMC3064346

[43] Rost B , Yachdav G, Liu J. 2004. The PredictProtein server. Nucleic Acids Res. 32:W321–W326. 15215403 10.1093/nar/gkh377 PMC441515

[44] Lander GC , et al 2006. The structure of an infectious P22 virion shows the signal for headful DNA packaging. Science. 312:1791–1795. 16709746 10.1126/science.1127981

[45] Liu X , et al 2010. Structural changes in a marine podovirus associated with release of its genome into Prochlorococcus. Nat Struct Mol Biol. 17:830–836. 20543830 10.1038/nsmb.1823 PMC2924429

[46] Jiang W , et al 2006. Structure of epsilon15 bacteriophage reveals genome organization and DNA packaging/injection apparatus. Nature. 439:612–616. 16452981 10.1038/nature04487 PMC1559657

[47] Wilkinson ME , Kumar A, Casañal A. 2019. Methods for merging data sets in electron cryo-microscopy. Acta Crystallogr D Struct Biol. 75:782–791. 31478901 10.1107/S2059798319010519 PMC6719665

[48] Punjani A , Rubinstein JL, Fleet DJ, Brubaker MA. 2017. Brubaker, cryoSPARC: algorithms for rapid unsupervised cryo-EM structure determination. Nat Methods. 14:290–296. 28165473 10.1038/nmeth.4169

[49] Pettersen EF , et al 2021. UCSF ChimeraX: structure visualization for researchers, educators, and developers. Protein Sci. 30:70–82. 32881101 10.1002/pro.3943 PMC7737788

[50] Scheres SHW . 2012. RELION: implementation of a Bayesian approach to cryo-EM structure determination. J Struct Biol. 180:519–530. 23000701 10.1016/j.jsb.2012.09.006 PMC3690530

[51] Jumper J , et al 2021. Highly accurate protein structure prediction with AlphaFold. Nature. 596:583–589. 34265844 10.1038/s41586-021-03819-2 PMC8371605

[52] Emsley P , Lohkamp B, Scott WG, Cowtan K. 2010. Features and development of coot. Acta Crystallogr D Biol Crystallogr. 66:486–501. 20383002 10.1107/S0907444910007493 PMC2852313

[53] Croll TI . 2018. ISOLDE: a physically realistic environment for model building into low-resolution electron-density maps. Acta Crystallogr D Struct Biol. 74:519–530. 29872003 10.1107/S2059798318002425 PMC6096486

[54] Cianfrocco MA , Wong-Barnum M, Youn C, Wagner R, Leschziner A. 2017. COSMIC2: A science gateway for cryo-electron microscopy structure determination. Proceedings of the practice and experience in advanced research computing 2017 on sustainability, success and impact. New Orleans (LA), USA: Association for Computing Machinery. p. Article 22.

[55] Waterhouse A , et al 2018. SWISS-MODEL: homology modelling of protein structures and complexes. Nucleic Acids Res. 46:W296–W303. 29788355 10.1093/nar/gky427 PMC6030848

[56] Williams CJ , et al 2018. MolProbity: more and better reference data for improved all-atom structure validation. Protein Sci. 27:293–315. 29067766 10.1002/pro.3330 PMC5734394

