# Supplementary material for: Ejectosome of Pectobacterium bacteriophage ΦM1
Source: PNAS Nexus. 2024 Sep 19;3(9):pgae416. doi: 10.1093/pnasnexus/pgae416 (PMC11440229; doi:10.1093/pnasnexus/pgae416)
Supplement: pgae416_Supplementary_Data [file pgae416_supplementary_data.zip › PNASNEXUS-PNASNEXUS-2024-01075-TR-s01.pdf]

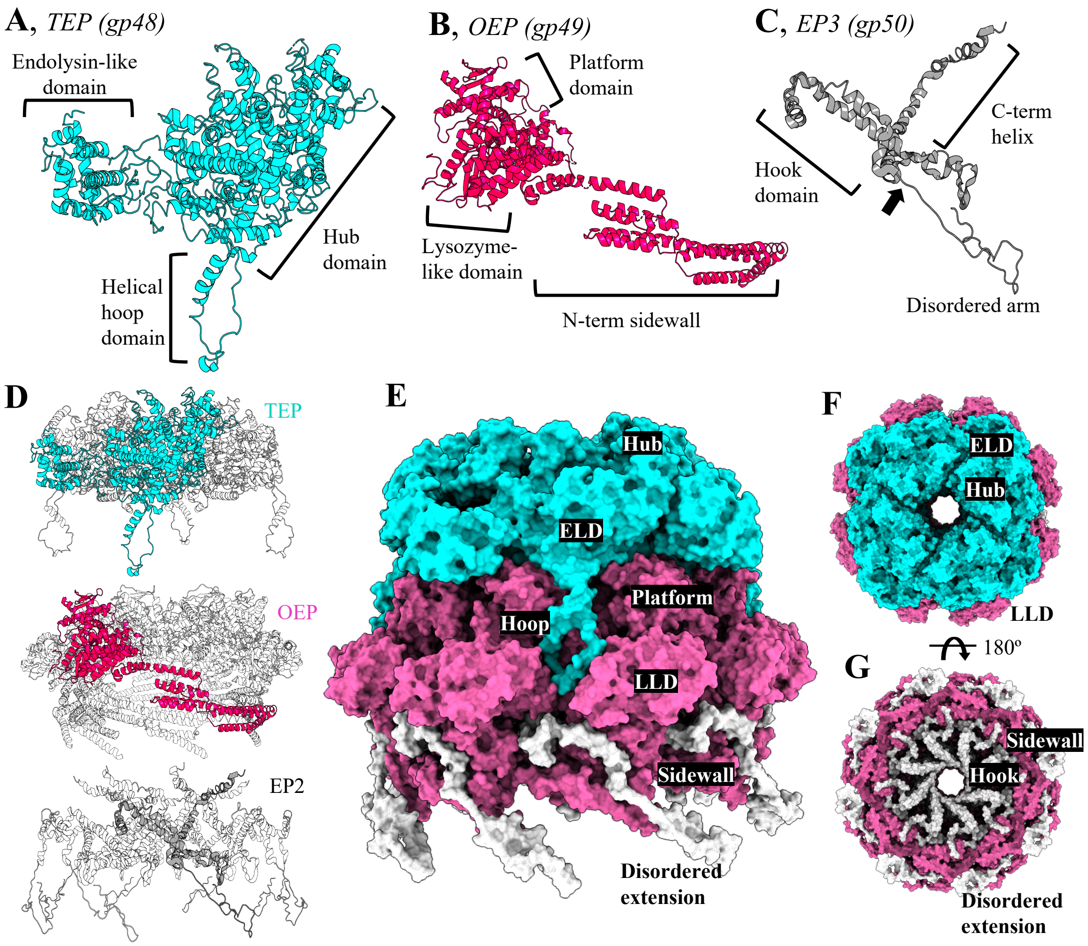

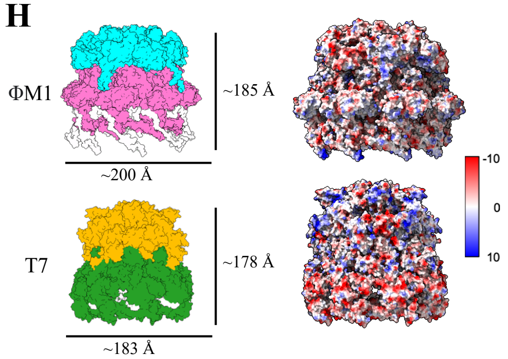

	ΦM1	T7
Total molecular weight	1.5 MDa	1.4 MDa
Buried surface area	179,820 Å^2	112,795 Å^2
Surface area	535,560 Å^2	404,859 Å^2

Fig. S3. ΦM1 ejectosome. Subdomains of tetrameric ejection protein (*a*), octameric ejection protein (*b*) and EP3 (*c*) are labelled. Density in the EP3 cryo-EM map terminates near the arrow. The proposed structure of the N-terminal disordered extension is derived from AlphaFold and is preserved in this figure to give the reader an indication of the true length of the ejectosome assembly. (*d*) One chain of the TEP, OEP and EP3 is coloured, with symmetry mates of the respective assemblies displayed as silhouettes. (*e*) Molecular surface of the ejectosome is displayed from the side with domains labelled. (*f*) A view of the top of the ejectosome looking down the DNA channel shows the grooves in the hub which seat the DNA as it enters the tail assembly. (*g*) A bottom view shows the C8 arrangement of the EP3 hooks which interfaces with the top of the portal assembly (not shown). (*h*) A size comparison of ΦM1 ejectosome and T7 (7EYB) shows ΦM1 is both wider and taller. The electrostatic potential of the ejectosome assemblies are shown

to the right. Note, the EP3 model from T7 was too short to produce a meaningful overlay. RMSD was calculated in UCSF ChimeraX. A DALI structure database search was performed for the ELD, hub, platform, sidewall and LLD to probe for similar protein structures. DALI returned similar matches only for the endolysin-like domain of TEP and the lysozyme-like domain of OEP, matching with endolysin and lysozyme, respectively. Buried surface area and surface area were calculated in PDBePISA.

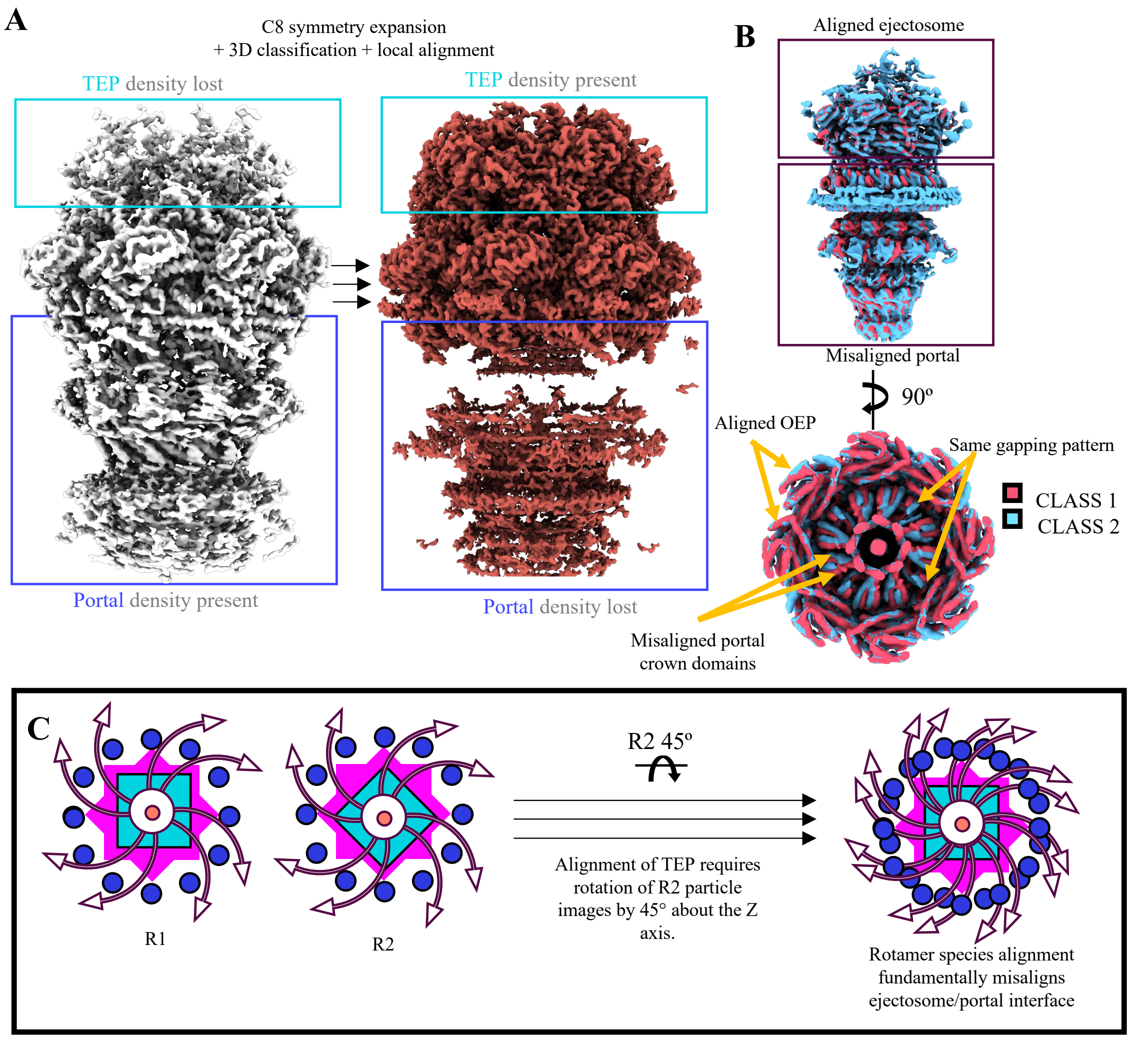

Fig. S4. Evidence for the presence of ejectosome rotamer species. (*a*) Ejectosome orientations were established by first aligning portal images, followed by a box shift in Z, and C12 symmetry expansion + 3D classification to find particle orientations with the portal correctly aligned to the ejectosome. TEP density was missing as shown in the cyan box. Portal density was visible but smeared; this is suspected to occur due to the presence of portal rotamer species, in which the portal rotates into two positions separated by 6° about the Z axis (below). C8 symmetry expansion and 3D classification refined particle orientations such that density corresponding to the TEP became visible at the expense of density corresponding to the portal. Both reconstructions represented were resolved using C4 symmetry. (*b*) Two representative 3D classes that show portals rotated by 6° relative to the ejectosome. (c) Theoretical depiction of how the presence of TEP rotamer species fundamentally prevents alignment of the portal and full ejectosome using a complete particle stack. Two rotamer species are depicted as R1 and R2; each species is identical except with the exception that TEP tetramers are rotated 45° about the Z axis relative to each other.

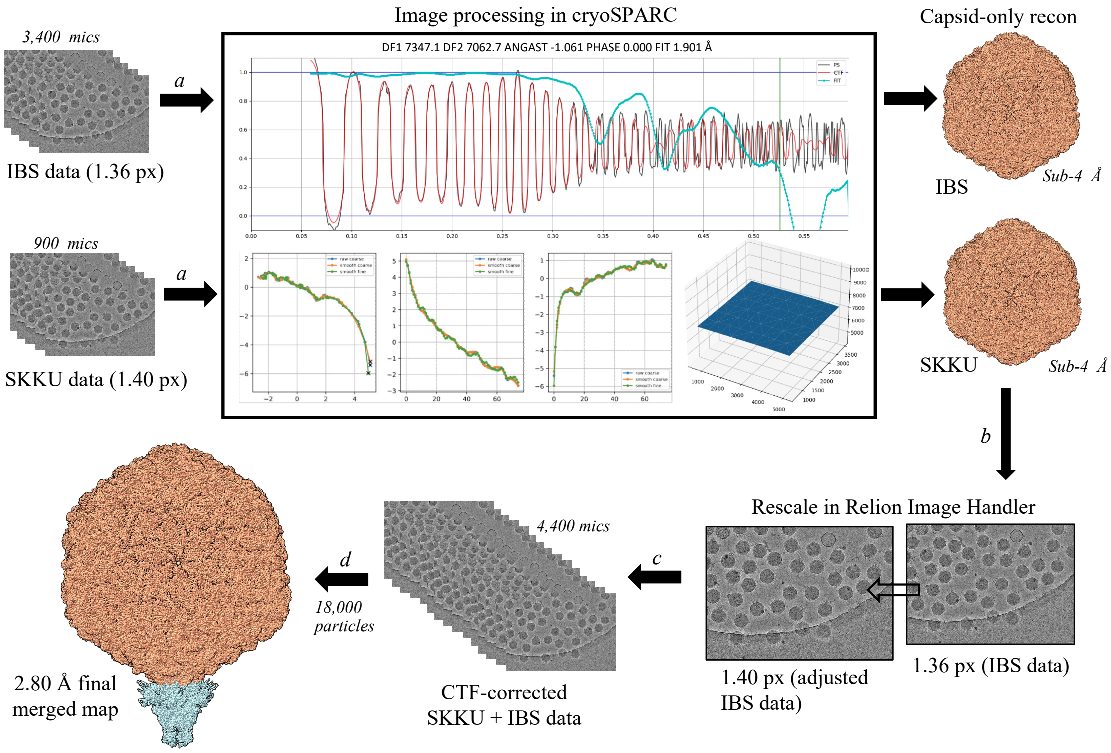

Fig. S5. Data merging. Two datasets with different pixel sizes (IBS, 1.36; SKKU, 1.40) were image processed (*a*) in cryoSPARC v3 by patch-based motion correction, patch-based CTF correction, automated particle picking, *ab initio* reconstruction and homogeneous refinement. Two capsid maps were subsequently produced with different voxel sizes. The maps were imported into UCSF ChimeraX (*b*) and assessed for cross-correlation before the IBS micrographs were imported into Relion Image Handler and rescaled to the same pixel size as the SKKU data using the Rescale function. The data (*c*) were merged in cryoSPARC by correcting the CTF separately and merging the particles stacks at the 2D classification stage. Standard reconstruction methods were then applied as described in methods section 5 and detailed in Fig. S2 and S3. Finally, consensus maps for the capsid and tail (*d*) were obtained from the information contained within both datasets and merged to form a complete phage reconstruction. Methods adapted from Wilkinson *et al.,* **(2).**

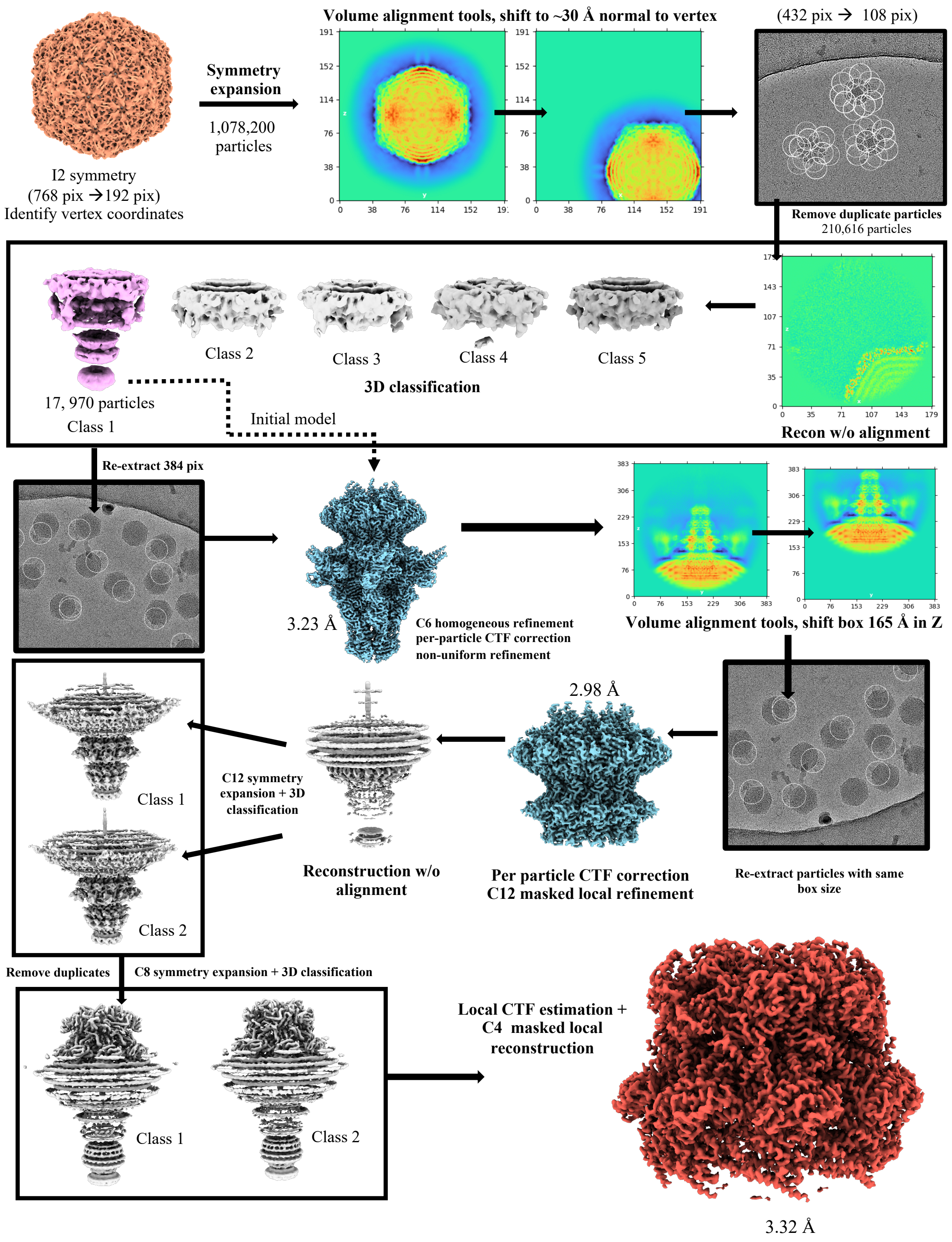

Fig. S6. Reconstruction approach for tail and ejectosome assembly. See supplementary method 1 for a comprehensive protocol-style description of the workflow. Reconstruction workflow was followed in cryoSPARC v4.2.1. Initial vertex coordinates were established using supplied supplementary python script (script 1: script relies on I2 capsid reconstruction). Output maps were visualized in UCSF ChimeraX v1.6.

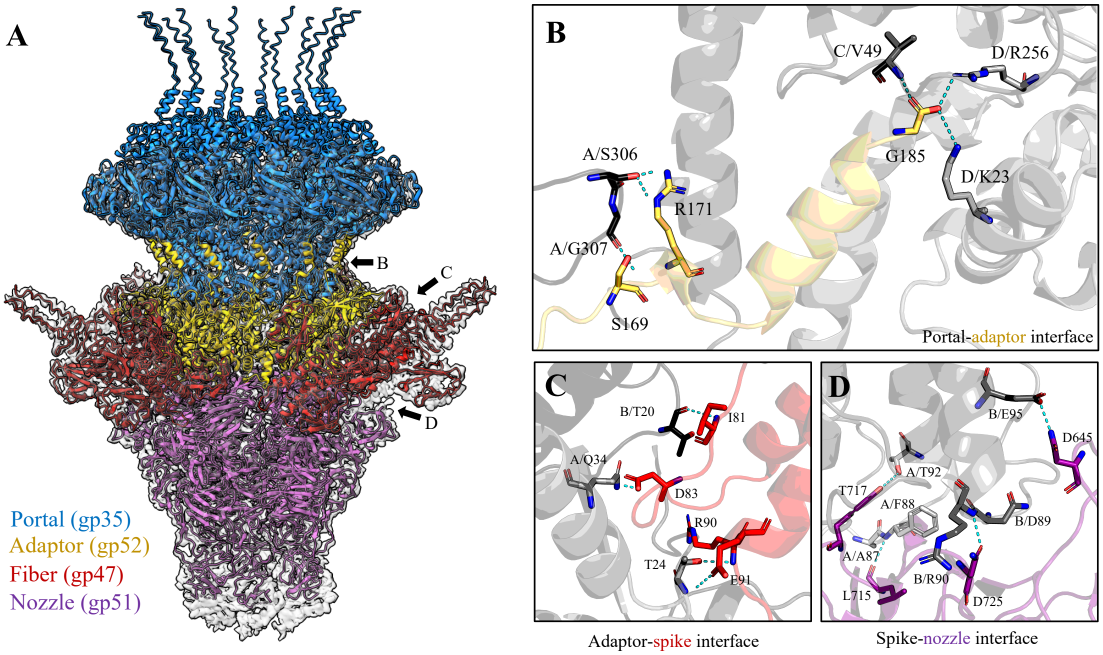

Fig. S7. ΦM1 portal and tail machinery interfaces. (*a*) The portal and tail machinery viewed from the side, displayed as Richardson ribbons nested within the preliminary tail density obtained at low resolution. Proteins and their gene protein identities are labelled. Map density shown was resolved using C6 symmetry. (*b*) The interface between the portal (shades of greys) and adaptor (yellow) involve three chains of gp35 and one chain of gp52; adaptor residue G185 interacts with two chains of gp35, while R171 and S169 interact with another chain of gp35. (*c*) The interface between the adaptor (now grey) and the spike (red) involves two adaptor chains to one spike. (*d*) The interface of the spike and nozzle.

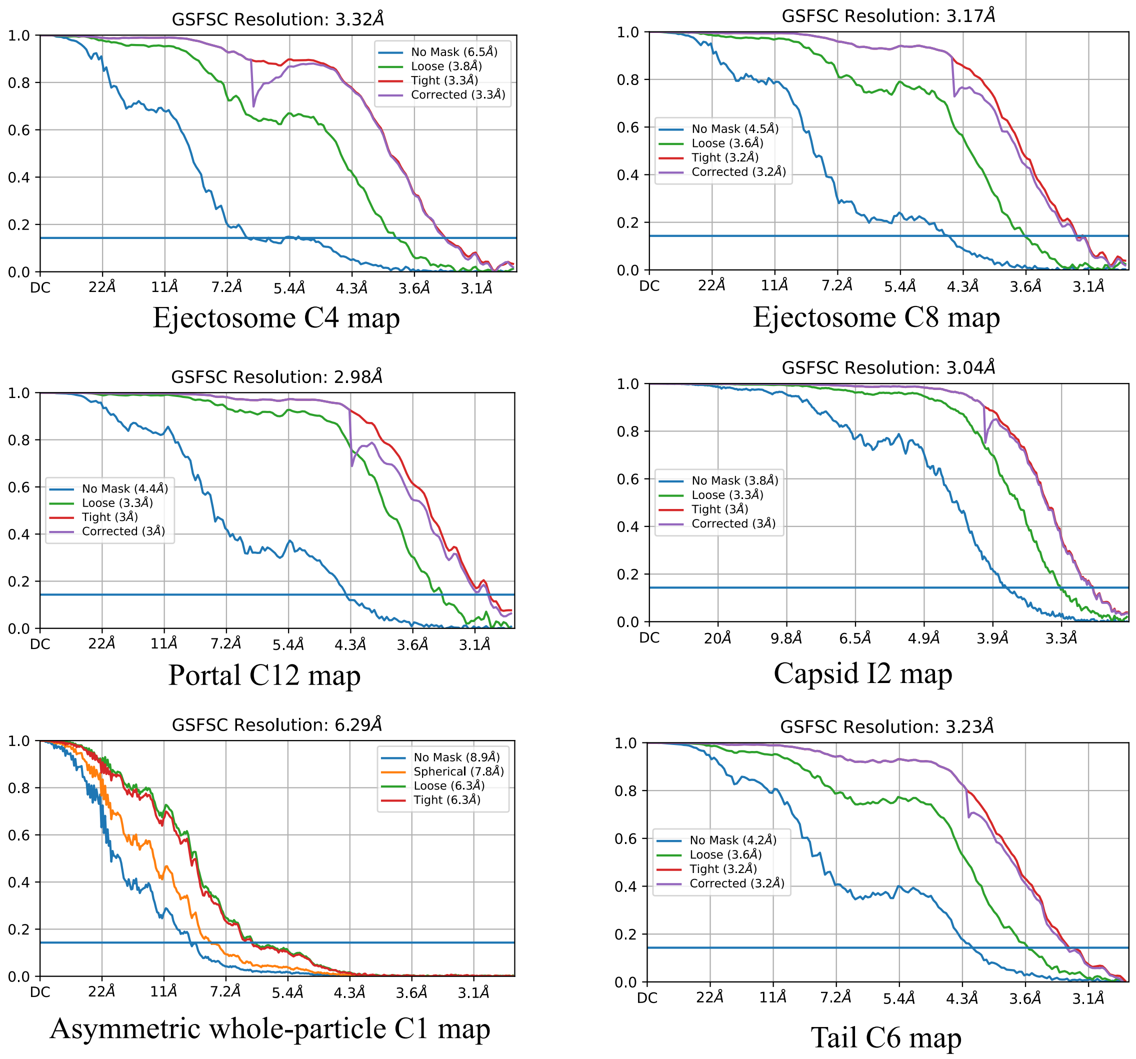

Fig. S8. Fourier Shell Correlation Plots for *Pectobacterium* phage ΦM1 maps. Plots were produced from non-uniform refinement jobs in cryoSPARC v4.2.1. The maps associated with each plot are listed beneath.
