# Supplementary material for: Ejectosome of Pectobacterium bacteriophage ΦM1
Source: PNAS Nexus. 2024 Sep 19;3(9):pgae416. doi: 10.1093/pnasnexus/pgae416 (PMC11440229; doi:10.1093/pnasnexus/pgae416)
Supplement: pgae416_Supplementary_Data [file pgae416_supplementary_data.zip › PNASNEXUS-PNASNEXUS-2024-01075-TR-s02.pdf]

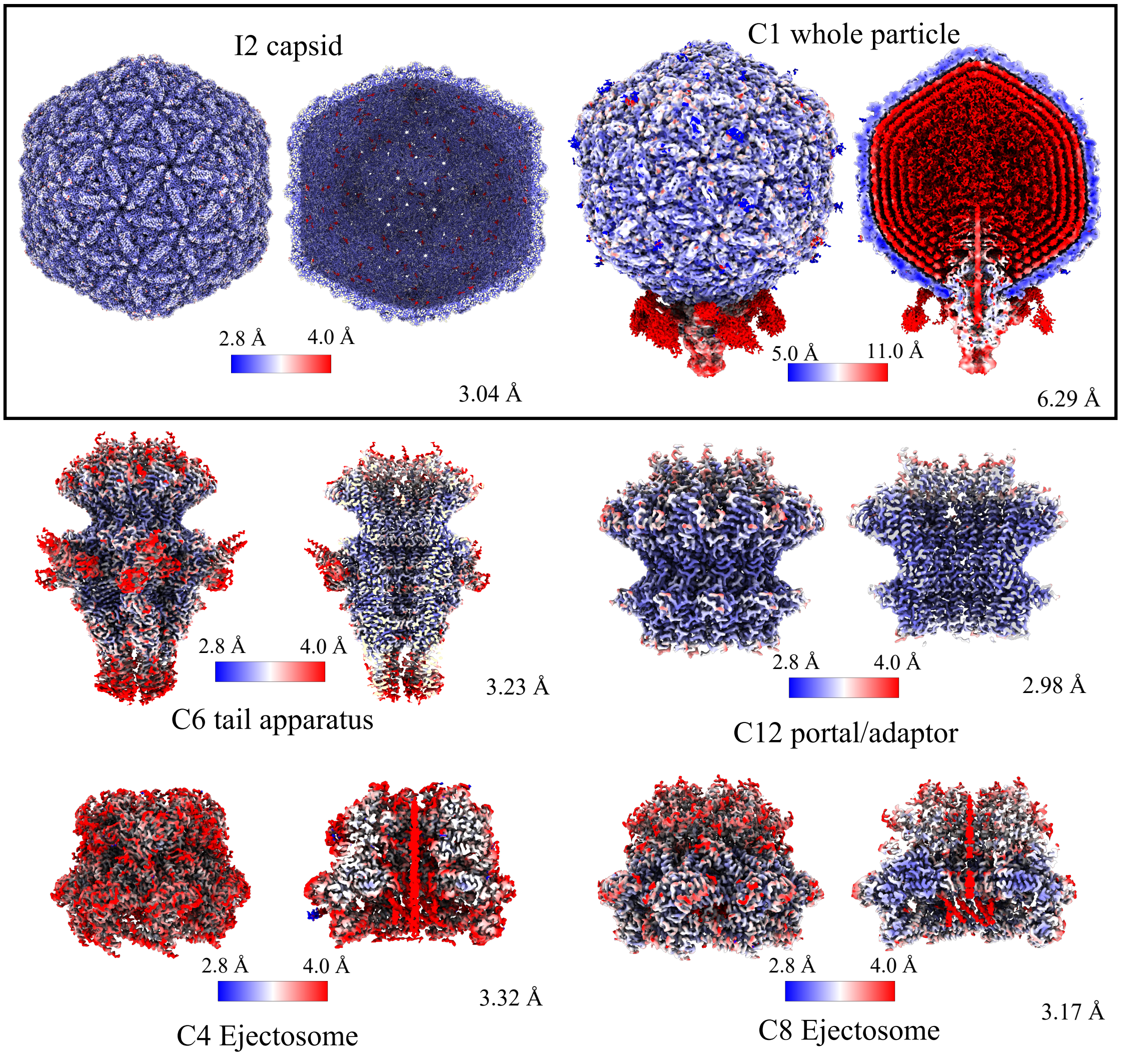

Fig. S9. Local resolution depiction of all deposited maps. I2 capsid contour threshold: 0.394 (PDB: 8VB0), C1 whole particle contour threshold: 0.119 (EMDB: EMD-43132), C6 spike-nozzle tail apparatus contour threshold: 0.332 (PDB: 8VBX), C12 portal-adaptor contour threshold: 0.443 (PDB: 8VB4), C4 ejectosome contour threshold: 0.205 (8VB2), C8 ejectosome contour threshold: 0.251 (EMDB: EMD-43111). Local resolution was calculated using cryoSPARC v4.2.1 Local Resolution job.

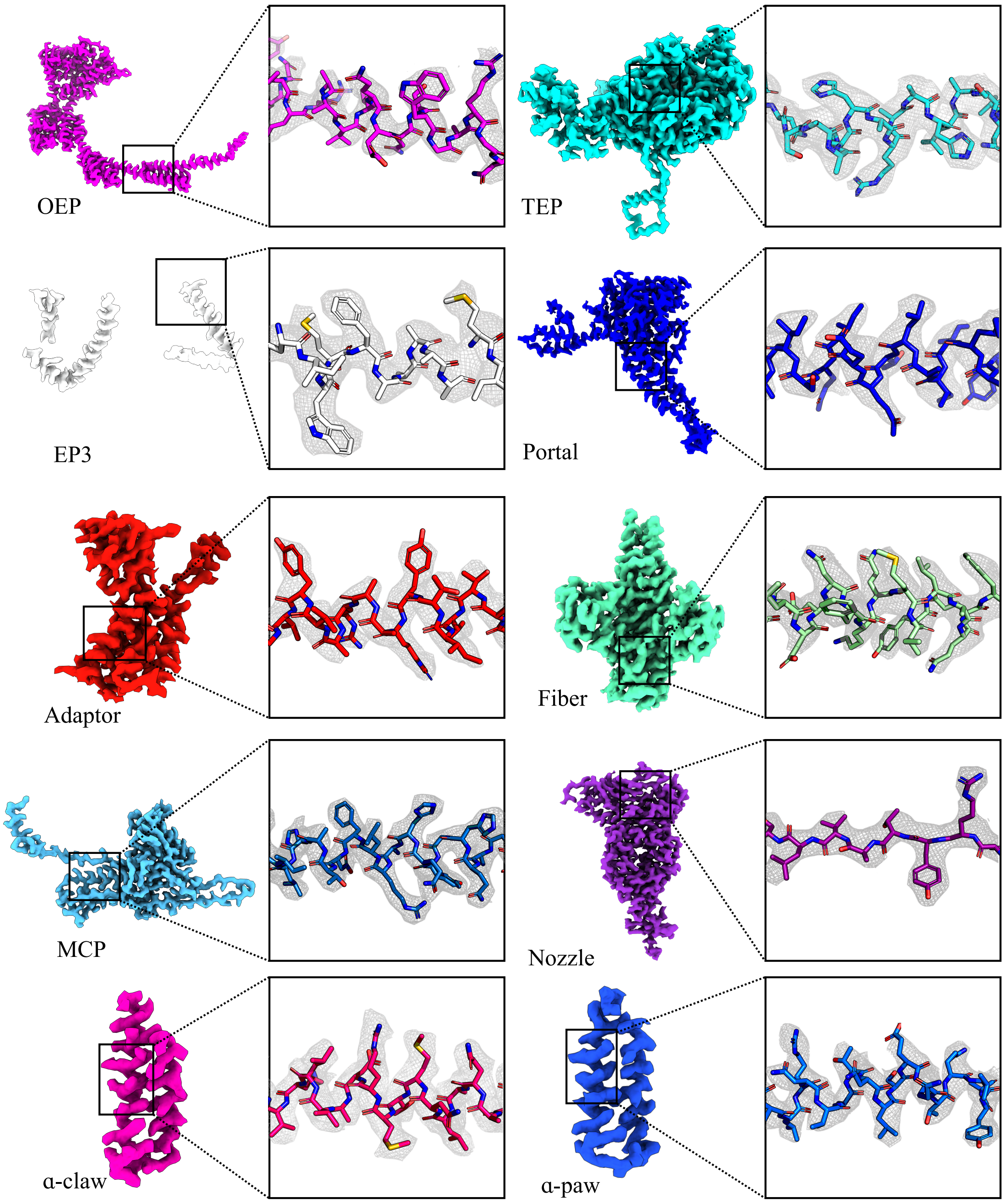

Fig. S10. Depiction of model map quality of fit for all deposited protein models. In each panel, left shows a zoned density corresponding to the protein (labeled below each panel). Right shows a representative helix from each model in stick representation and corresponding density. The nozzle lacks helices and so a representative beta strand is shown instead. Map contour levels are not displayed as all maps were boxed for visualization, resulting in modified density distribution.

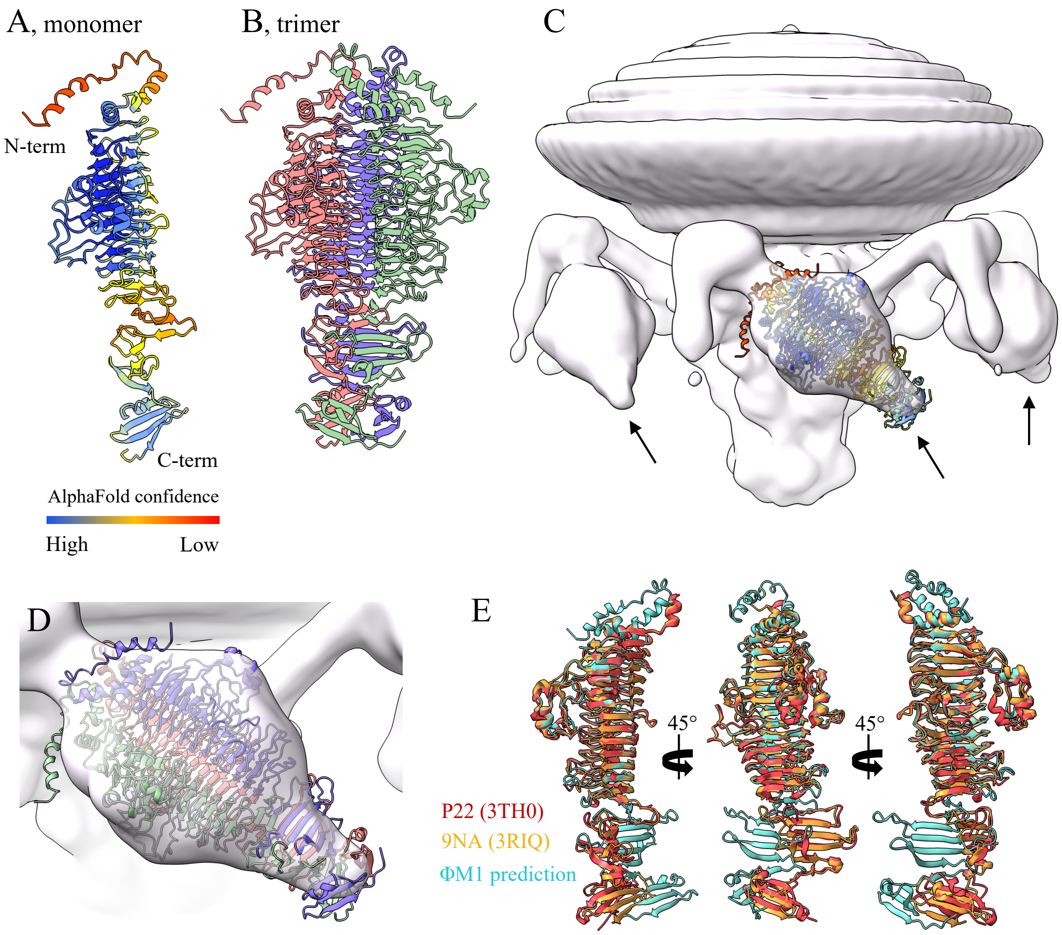

Fig. S11. Putative globular tail spike. The tail spike is likely encoded by gp39 of the ΦM1 genome, which is annotated in the NCBI description of the genome as 'tail spike protein'. Poor resolution of this domain precluded modelling. (*a*) An AlphaFold model of the protein (coloured by B factor) confidently predicts the core domain but loses confidence at the N- and C- termini. (*b*) The trimeric arrangement of gp39 broadly resembles the shape of the density identified for the spikes, seen in panel C marked by arrows. (*c*) A composite reconstruction of the tail spike with low-pass Gaussian filter applied. The globular domains of the tail spike density have clear 3-fold symmetric features even when reconstructed in C1, suggesting the spike is likely a trimer. The AlphaFold model coloured by B factor is fit within the filtered density with the C-terminus placed as the most distal domain. (*d*) The AlphaFold model of the gp39 is fit inside the low-pass filtered tail spike density. (*e*) A DALI structure search returned the Siphovirus 9NA tail spike receptor binding protein (PDB: 3RIQ) as the most structurally related PDB to the AlphaFold gp39 structure, followed by phage P22 tail spike (PDB: 3TH0). Overlays were produced in UCSF ChimeraX.

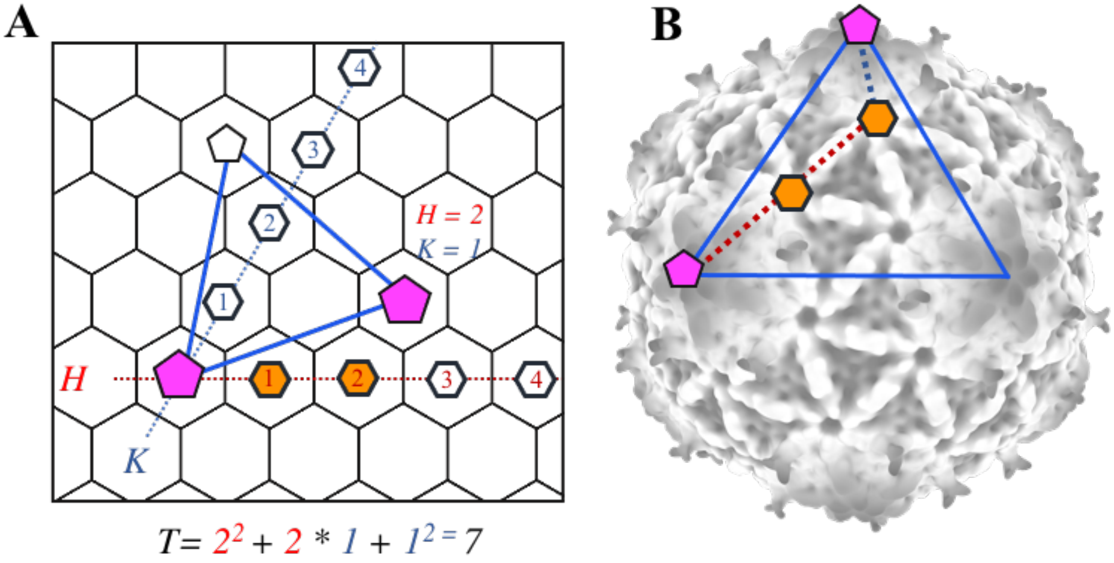

Fig. S12. Capsid triangulation. (*a*) Schematic explanation of capsid triangulation. Icosahedrons may be constructed by taking a planar hexagonal net and removing a 1/6th triangular wedge from hexagons, such that they become pentagonal vertices. This is iteratively repeated in triangular patterns to produce 20 triangular facets (a single example shown in dark blue) traced between pentagons, which can be folded into a faceted icosahedron with 12 vertices. The icosahedron can be made more complex, by increasing the number of hexagons between pentagons in a planar hexagonal net, such that the distance between pentagons is greater. This increasing distance can be described in terms of *H* and *K* which denote the number of hexagons and pentagon, measured from their centres, between two vertex pentagons of a singular facet, on skewed axes (H axes indicated with red dotted line, K axes in blue dotted line). Triangulation (T) can then be calculated by entering values of H and K into the triangulation equation: $T = H^2 + HK + K^2$. (*b*) practical application of triangulation to φM1. Pentamers are indicated with magenta pentagons. Hexamers are indicated with orange hexagons. A singular triangular facet is shown in dark blue. Lines corresponding to the 2D axes of H and K are coloured in red and blue according to panel A convention.

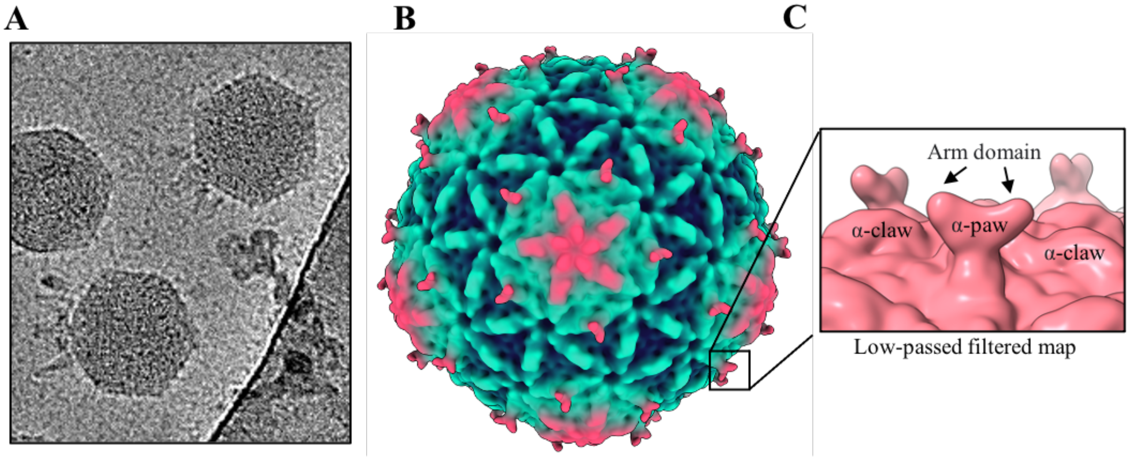

Fig. S13. ΦM1 capsid decoration proteins. (**A**) Cryo-EM micrograph depicting phage particles with an 8 Å low pass filter applied. Thin, hair-like projections can be seen protruding from the particles, corresponding to the long C-terminal fiber of the α-paw. (**B**) Icosahedral averaged capsid volume with a low-pass Gaussian filter applied in UCSF ChimeraX and coloured radially from dark blue (near center) to pink (extending outward). The pink projections correspond to the extended C-terminal arm domain of the α-paw. (**C**) A closer view of the low-pass filtered density of the α-paw shows two continuous densities for this extended domain, indicating the α-paw dimer may diverge into two independent protruding domains, or the C-terminus may have partial occupancy in two distinct conformations.

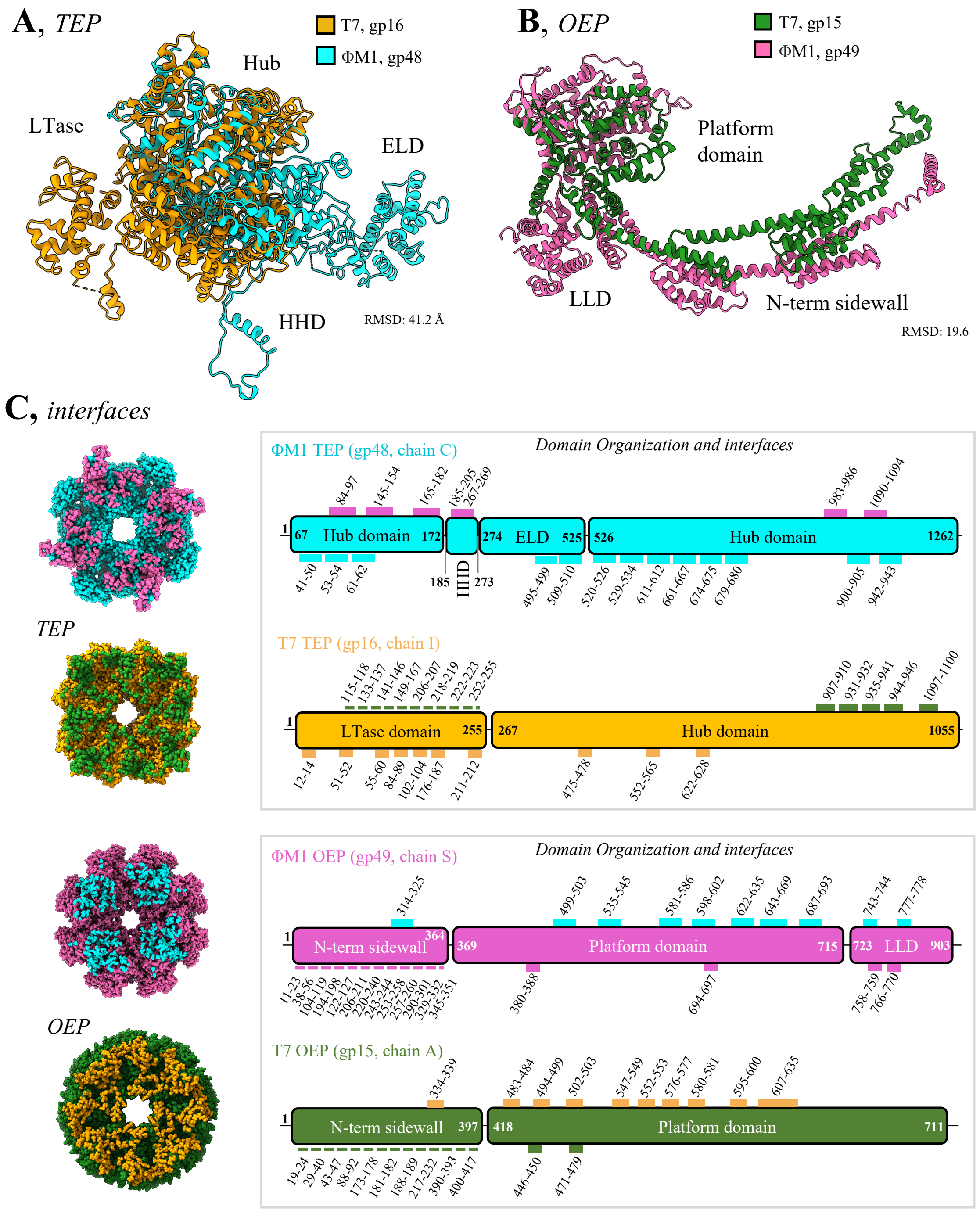

Fig. S14. Comparison of ΦM1 ejectosome with phage T7. A structural overlay of the TEP (*a*) from bacteriophage T7 (PDB: 7EYB) was performed against ΦM1 TEP and RMSD values are calculated against all atoms. The same is shown for the OEP (*b*). Note, the EP3 model from T7 was too short to produce a meaningful overlay. (*c*) Interfaces within the ΦM1 and T7 TEP and OEP are shown with residues within 5 Å displayed. A domain organization schematic of each protein is shown to the right. Amino acid numbers are listed within each domain. Protein interfaces larger than 2 aa (as calculated in PDBePISA) are shown as small squares above or below the protein with aa ranges provided. For visual simplicity, domains are not to scale. Interfaces are as follows;

ΦM1 TEP chain C against TEP chain B and OEP chain S; T7 TEP chain I against T7 TEP chain L and T7 OEP chain A; ΦM1 OEP chain S against ΦM1 TEP chain C and ΦM1 OEP chain R, and T7 OEP chain A against T7 TEP chain I and T7 OEP chain H. As a significant proportion of the EP3 is not modelled, EP3 was excluded from the interface analysis.

SI References

1. D. Liebschner *et al.*, Macromolecular structure determination using X-rays, neutrons and electrons: recent developments in Phenix. *Acta Crystallogr D Struct Biol* **75**, 861-877 (2019).
2. M. E. Wilkinson, A. Kumar, A. Casañal, Methods for merging data sets in electron cryo-microscopy. *Acta Crystallogr D Struct Biol* **75**, 782-791 (2019).
