# Supplementary material for: Ejectosome of Pectobacterium bacteriophage ΦM1
Source: PNAS Nexus. 2024 Sep 19;3(9):pgae416. doi: 10.1093/pnasnexus/pgae416 (PMC11440229; doi:10.1093/pnasnexus/pgae416)
Supplement: pgae416_Supplementary_Data [file pgae416_supplementary_data.zip › PNASNEXUS-PNASNEXUS-2024-01075-TR-s03.pdf]

PNAS nexus

Supporting Information for

Ejectosome of *Pectobacterium* bacteriophage ΦM1

Alice-Roza Eruera[†, 1], James Hodgkinson-Bean[†, 1], Georgia L. Rutter[†, 2], Francesca R. Hills[†, 3], Rosheny Kumaran[†, 3], Alexander J. M. Crowe[†, 3], Nickhil Jadav[†, 3], Fangfang Chang[†, 3], Klemens McJarrow-Keller[†, 3], Fátima Jorge[‡, 4], Jaekyung Hyun[§, 4], Hyejin Kim[#, 4], Bumhan Ryu[#, 4], Mihnea Bostina[†, 5, *]

[†]Department of Microbiology and Immunology, University of Otago, Dunedin, New Zealand

Alice-Roza Eruera, James Hodgkinson-Bean, Georgia L. Rutter, Francesca R. Hills, Rosheny Kumaran, Alexander J. M. Crowe, Nickhil Jadav, Klemens McJarrow-Keller, Fangfang Chang, Mihnea Bostina

[‡]Otago Micro and Nanoscale Imaging, University of Otago, Dunedin, New Zealand.

[§]School of Pharmacy, Sungkyunkwan University (성균관대학교), Suwon, South Korea

Jaekyung Hyun

[#]Institute for Basic Science (기초과학연구원), Daejeon, South Korea

Hyejin Kim, Bumhan Ryu

Corresponding author: Mihnea Bostina
Email: mihnea.bostina@otago.ac.nz

This PDF file includes:

Supporting text
Figures S1 to S14
Tables S1 to S7
SI References

Supporting Information Text

Supplementary method 1. CryoSPARC-based reconstruction protocol for resolving ejectosomes from podophages.

Prerequisites
Phage tail particle coordinates (e.g., a particle stack of tail picks)
UCSF ChimeraX v2 or better
Any version of cryoSPARC up to at least version 4 (most current version at time of publication)

Foreword
This protocol is intended for use in cryoSPARC but the principles should be broadly applicable for any cryo-EM processing software of choice. This protocol first assumes a DNA-full phage tail class has already been obtained by either Homogeneous Refinement or Non-Uniform refinement. Unless otherwise specified, job types are run with default parameters (although the investigator is encouraged to change parameters important to their specimen of interest).

Protocol
To resolve an ejectosome, particle coordinates need to first be realigned so that the *center coordinate* of the particles are over the general location of the *ejectosome* (and not in the center of the *tail*). After this, reconstruction methods are relatively standard.

1. Obtain a reconstruction of the tail apparatus via Homogeneous Refinement or Non-Uniform Refinement.
    i. Note: It is important that symmetry is imposed in this reconstruction. Symmetry imposed may be either C6 or C12; the important criteria is that a high-quality portal structure is present in the tail reconstruction.
2. Import the particle coordinates from either refinement job into the Volume Realignment Tools job in cryoSPARC.
3. Shift the particle box along the Z axis so that the center of the particle box is roughly centered over the expected location of the ejectosome (i.e. within the capsid, above the portal).
    i. Note: The distance to shift will depend on the specimen being reconstructed and may require iterative optimization. In the case of bacteriophage ΦM1, shifting 165 Å along the Z axis worked well. We recommend starting with 165 Å as a preliminary value.
4. Import the new, realigned particle coordinates from the Volume Realignment Tools job into an Extract Particles job. Extract using appropriate parameters for your specimen.
    i. Note: The box size will need to be large enough to contain the ejectosome but small enough that minimal DNA is included within the box. DNA is a high-contrast, poorly ordered molecule and will overpower the weight of the ejectosome when the particles are aligned in downstream steps. Running a few Extract Particles jobs with progressively smaller box sizes may be beneficial, until an appropriate size is found which contains the ejectosome but minimal DNA.)
5. Submit the particle stack from the Extract Particles job (step 4) to a C12 Symmetry Expansion job.
    i. Note: At this point, masked local reconstructions of the ejectosome region will likely yield poor results, as tails will be aligned such that the ejectosome is rotated in several symmetry-related positions relative to the portal, resulting in disordered density. Symmetry Expansion allows re-insertion of each particle image into the Fourier shell in all symmetry-related positions. Then, by focused 3D Classification, particle images that have ejectosomes aligned in the same orientation may be pooled for further refinements.
6. Import the output from the Symmetry Expansion job into a 3D Classification job and run it with default parameters.

i. We strongly recommend the use of a soft focus mask centered on the ejectosome region. A general blob shaped mask worked well in the case of ΦM1. (For soft mask generation, the investigator is directed to the cryoSPARC mask guide; https://guide.cryosparc.com/processing-data/tutorials-and-case-studies/mask-selection-and-generation-in-ucsf-chimera.)
ii. If <1,000,000 particles are available, we recommend selecting for no more than 3-5 classes. If >1,000,000 particles are available, expanding the search range through more classes may be beneficial. As there are 3 symmetry-related positions available for the ejectosome, normally 3 good classes should emerge, each ~4 times the size of the original particle stack.

7. Inspect the output volumes from the 3D Classification job. A 'good' class should contain some helices corresponding to the octahedral components near the portal.

8. Run a Remove Duplicate Particles job on any of the 'good' classes. The particle stack size should now be roughly equal to the initial particle stack size.

9. Submit the particles from the Remove Duplicate Particles job to a C8 Symmetry Expansion job.

10. Import the particle stack from the C8 Symmetry Expansion job to a 3D Classification job, with the same number of classes as in step 8 (again, a focus mask is recommended).

i. Note: Here we expect *two* good classes, as tetrameric ejectosome components may sit in two symmetry-related positions on top of the octameric region.

11. Inspect the output volumes from the 3D Classification job.

12. Either good class should contain a fully aligned ejectosome. This volume can be used as an initial model, and/or as a template for local mask generation, in focused local refinement (after running a Remove Duplicates job). We found masked focused local refinement (without particle subtraction) worked best, presumably as DNA is excluded during refinement.

Table S1. Sample preparation and data collection statistics.

		Sungkyunkwan University data set	Institute for Basic Sciences data set
Grid preparation	EM-Grid	Quantifoil R1.2/1.3 Cu300	Quantifoil R1.2/1.3 Cu300
	Glow discharge (GD)	Negative	Negative
	GD current	15 mA	15 mA
	GD time	30 seconds	30 seconds
	GD pressure	0.39 mbar	0.39 mbar
Vitrification	Loading volume	4 µL	4 µL
	Loading side	Carbon	Carbon
	Blot time	5 seconds	3 seconds
	Blot force	0	0
	Temp/humidity	4°/100%	4°/90%
	Wait time	0 seconds	10 seconds
Microscope	Model	Krios G4	TFS Krios G4
	Spherical Aberration; Cs [mm]	2.7	2.7
	Dose rate [e/pix/s]	8	8.3
	Pixel value [Å/pix]	1.4	1.36
	Nominal Magnification	64,000 X	64,000 X
	Calibrated Magnification	36,765 X	36,765 X
	Exposure time [sec]	12.25	12.02
	# of Fractions	50	50
	Condenser lens aperture [µm]	70	70
	Objective lens aperture [µm]	100	100
	Defocus range (step size)	-0.8, -1.0, -1.2, -1.5, -1.8, -2.1, -2.4	-0.8, -1.0, -1.2, -1.5, -1.8, -2.1, -2.4
	Total dose [e/Å^2]	53.38	53.9
	Dose per fraction [e/Å^2/Frac.]	1.07	1.07
	AFIS (Fast Mode)	None	None
	Alpha Tilt [degrees]	0	0

Data	Model	Gatan K3 BioContinuum	Gatan K3 BioContinuum
	Energy slit width	20 eV	20 eV
	Acquisition mode	EC	EC
	Correlated double sampling (CDS) mode	Used	Used
	Movie format	Tiff(LZW)	Tiff(LZW)
	Gain normalization	No	No

Table S2. Reconstructed protein components of bacteriophage ΦM1. The colours are consistent with figure elements and representations shown in this paper. All reconstructed proteins are listed. All molecular weights (MW) were estimated in ExPasy based on the amino acid sequence (https://web.expasy.org/protparam/). In the case of gp43 and gp44, the assembly is a dimer. The NCBI Taxonomy ID for bacteriophage ΦM1 is 1211386 and GenBank accession number YP_009591954.1. A single phage particle is composed of 23.8 Mda of protein.

Protein name	Gp ID	Symmetry group	MW (monomer)	MW (assembly)	Monomers per phage (n = 909)
Major capsid protein	gp38	I,222r	36.4 kDa	15 MDa	415
α-paw decoration	gp43	N/A	17 kDa	34 kDa (per dimer)	118
α-claw decoration	gp44	N/A	6 kDa	12 kDa (per dimer)	290
Tetrameric ejection protein (TEP)	gp48	C4	135.2 kDa	540.8 kDa	4
Octameric ejection protein (OEP)	gp49	C8	97.9 kDa	783.2 kDa	8
Ejection protein 3	gp50	C8	21.5 kDa	172 kDa	8
Portal protein	gp35	C12	55.6 kDa	667.2 kDa	12
Adaptor protein	gp52	C8	21.1 kDa	253.2 kDa	12
Fiber	gp47	C6	55.8 kDa	167.4 kDa (trimer)	18
Nozzle protein	gp51	C6	84.4 kDa	506.4 kDa	6

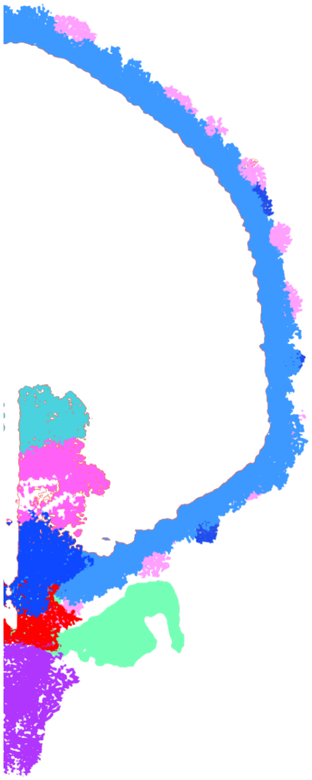

Tables S3 and S4. (1) Validation table for deposited capsid asymmetric unit model (inclusive of MCP, α-claw and α-paw proteins). (2) Validation table for deposited C6 tail model (inclusive of tail nozzle and tail fibre proteins). Validation tables were generated using Phenix (1).

**Model: Asymmetric unit of bacteriophage PhiM1 mature capsid**	
**Refinement map resolution:** 3.04 Å	
**Composition (#)**	
Chains	14
Atoms	20817 (Hydrogens: 0)
Residues	Protein: 2692 Nucleotide: 0
Water	0
Ligands	0
**Bonds (RMSD)**	
Length (Å) (# > 4σ)	0.004 (0)
Angles (°) (# > 4σ)	0.948 (1)
**MolProbity score**	**1.38**
**Clash score**	**3.42**
**Ramachandran plot (%)**	
Outliers	0.00
Allowed	3.68
Favored	96.32
**Rama-Z (Ramachandran plot Z-score, RMSD)**	
whole (N = 2664)	-0.74 (0.16)
helix (N = 732)	1.03 (0.20)
sheet (N = 519)	-0.50 (0.21)
loop (N = 1413)	-1.32 (0.16)
**Rotamer outliers (%)**	**0.05**
**Cβ outliers (%)**	**0.00**
**Peptide plane (%)**	
Cis proline/general	0.0/0.0
Twisted proline/general	0.0/0.0
**CaBLAM outliers (%)**	**1.97**
**ADP (B-factors)**	
**Iso/Aniso (#)**	**20817/0**
**min/max/mean**	
Protein	9.65/84.19/38.15
Nucleotide	---
Ligand	---
Water	---
**Occupancy**	
**Mean**	**1.00**
**occ = 1 (%)**	**100.00**
**0 < occ < 1 (%)**	**0.00**
**occ > 1 (%)**	**0.00**
**Model vs. Data**	
CC (mask)	0.89
CC (box)	0.46
CC (peaks)	0.33
CC (volume)	0.80
Mean CC for ligands	---

**Model: C6 nozzle and fibre complex of the mature bacteriophage PhiM1 particle**	
**Refinement map resolution:** 3.23 Å	
**Composition (#)**	
Chains	24
Atoms	50010 (Hydrogens: 0)
Residues	Protein: 6510 Nucleotide: 0
Water	0
Ligands	0
**Bonds (RMSD)**	
Length (Å) (# > 4σ)	0.003 (0)
Angles (°) (# > 4σ)	0.946 (0)
**MolProbity score**	**1.72**
**Clash score**	**7.54**
**Ramachandran plot (%)**	
Outliers	0.00
Allowed	4.42
Favored	95.58
**Rama-Z (Ramachandran plot Z-score, RMSD)**	
whole (N = 6378)	-1.42 (0.10)
helix (N = 630)	0.28 (0.22)
sheet (N = 1830)	-0.50 (0.12)
loop (N = 3918)	-1.41 (0.09)
**Rotamer outliers (%)**	**0.00**
**Cβ outliers (%)**	**0.00**
**Peptide plane (%)**	
Cis proline/general	0.0/0.0
Twisted proline/general	0.0/0.0
**CaBLAM outliers (%)**	**2.50**
**ADP (B-factors)**	
**Iso/Aniso (#)**	**50010/0**
**min/max/mean**	
Protein	40.26/119.97/65.67
Nucleotide	---
Ligand	---
Water	---
**Occupancy**	
Mean	1.00
occ = 1 (%)	100.00
0 < occ < 1 (%)	0.00
occ > 1 (%)	0.00
**Model vs. Data**	
CC (mask)	0.85
CC (box)	0.63
CC (peaks)	0.53
CC (volume)	0.83
Mean CC for ligands	---

Tables S5 and S6. (1) Validation table for deposited C12 tail components (inclusive of portal and adaptor proteins). (2) Validation table for deposited ejectosome model (inclusive of TEP, OEP and EP3 proteins). Validation tables were generated using Phenix (1).

Model: C12 portal and adaptor complex of the mature bacteriophage PhiM1 particle

Refinement map resolution: 2.98 Å

**Composition (#)**	
Chains	24
Atoms	60348 (Hydrogens: 0)
Residues	Protein: 7848 Nucleotide: 0
Water	0
Ligands	0
**Bonds (RMSD)**	
Length (Å) (# > 4σ)	0.004 (0)
Angles (°) (# > 4σ)	0.909 (0)
**MolProbity score**	**1.47**
**Clash score**	**7.37**
**Ramachandran plot (%)**	
Outliers	0.00
Allowed	2.31
Favored	97.69
**Rama-Z (Ramachandran plot Z-score, RMSD)**	
whole (N = 7776)	1.19 (0.10)
helix (N = 3732)	2.15 (0.09)
sheet (N = 1032)	-0.32 (0.15)
loop (N = 3012)	-0.52 (0.12)
**Rotamer outliers (%)**	**0.00**
**Cβ outliers (%)**	**0.00**
**Peptide plane (%)**	
Cis proline/general	0.0/0.0
Twisted proline/general	0.0/0.0
**CaBLAM outliers (%)**	**2.02**
**ADP (B-factors)**	
**Iso/Aniso (#)**	**60348/0**
**min/max/mean**	
Protein	29.51/107.11/62.21
Nucleotide	---
Ligand	---
Water	---
**Occupancy**	
**Mean**	**1.00**
**occ = 1 (%)**	**100.00**
**0 < occ < 1 (%)**	**0.00**
**occ > 1 (%)**	**0.00**
**Model vs. Data**	
CC (mask)	0.82
CC (box)	0.59
CC (peaks)	0.54
CC (volume)	0.80
Mean CC for ligands	---

Model: C4 pre-infection ejectosome of the mature bacteriophage PhiM1 particle

Refinement map resolution: 3.32 Å

**Composition (#)**	
Chains	20
Atoms	92008 (Hydrogens: 0)
Residues	Protein: 12164 Nucleotide: 0
Water	0
Ligands	0
**Bonds (RMSD)**	
Length (Å) (# > 4σ)	0.004 (0)
Angles (°) (# > 4σ)	0.926 (0)
**MolProbity score**	**1.47**
**Clash score**	**8.04**
**Ramachandran plot (%)**	
Outliers	0.03
Allowed	2.10
Favored	97.87
**Rama-Z (Ramachandran plot Z-score, RMSD)**	
whole (N = 11980)	0.73 (0.08)
helix (N = 8140)	1.04 (0.06)
sheet (N = 460)	-0.61 (0.22)
loop (N = 3380)	-0.55 (0.11)
**Rotamer outliers (%)**	**0.00**
**Cβ outliers (%)**	**0.00**
**Peptide plane (%)**	
Cis proline/general	0.0/0.0
Twisted proline/general	0.0/0.0
**CaBLAM outliers (%)**	**2.03**
**ADP (B-factors)**	
**Iso/Aniso (#)**	**92008/0**
**min/max/mean**	
Protein	48.13/156.58/83.90
Nucleotide	---
Ligand	---
Water	---
**Occupancy**	
Mean	1.00
occ = 1 (%)	100.00
0 < occ < 1 (%)	0.00
occ > 1 (%)	0.00
**Model vs. Data**	
CC (mask)	0.84
CC (box)	0.58
CC (peaks)	0.54
CC (volume)	0.83
Mean CC for ligands	---

Table S7. Summary of missing residues for all protein chains modelled. Reported residues here indicate which residues have been resolved in at least one protein chain. In rare cases, side chains have been removed if electrostatic potential density was considered insufficient for placement, resulting in some main chain breaks.

Protein	Chain length	Modelled residues	Modelled (%)
MCP	327	2-327	99.7
α-claw	62	2-62	98.4
α-paw	179	2-58	31.8
Nozzle	776	2-142, 265-271, 276-776	83.4
Fiber	534	1-154	28.8
Adaptor	185	1-185	100.0
Portal	503	7-349, 359-484	96.6
EP3	204	1-29, 31-55, 93-140, 146-168	77.0
TEP	904	49-771, 783-903	93.3
OEP	1263	38-62, 67-172, 185-280, 288-309, 311-321, 356-361, 367-427, 446-493, 496-506, 515-788, 820-1262	86.5

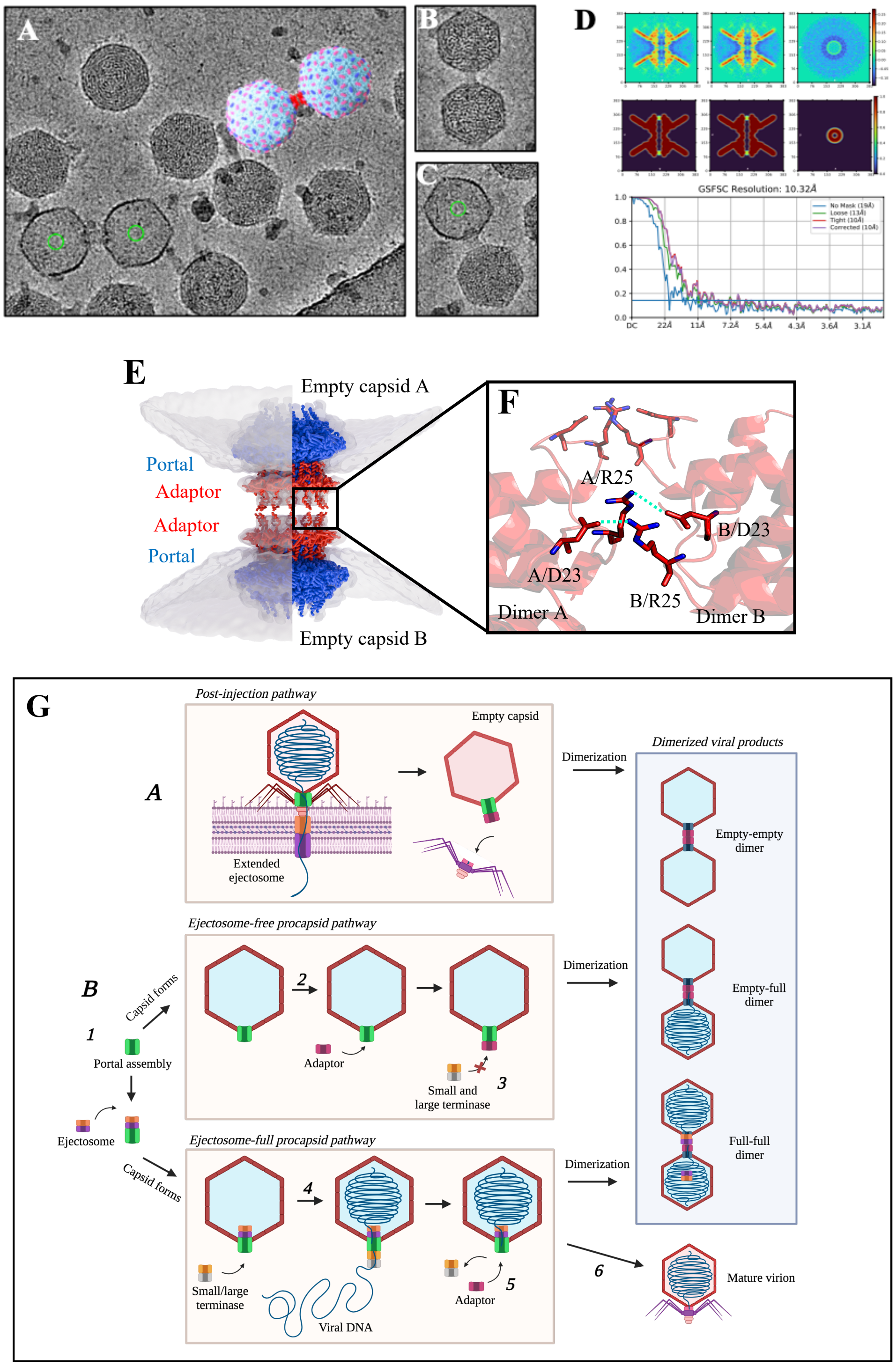

Fig. S1. Immature dimerized ΦM1 products. (*a*) Cryo-EM micrographs of three classes of immature capsid-like dimers. An artistic 3D render of the capsid dimer is placed over a dimer in the micrograph. The green circle indicates the center of a particle pick. Dimers can also be composed of two full (*b*) or empty-to-full capsids (*c*), but neither could not be resolved due to low particle counts (<60 particles each). Each class of capsid dimers (empty-empty, empty-full and full-full) were manually counted in the 3500+ micrographs of the merged data set. The empty-empty dimers were the most common product observed in these data. (*d*) Real-space slices and the gold-

standard Fourier shell correlation plots for the empty-empty structure produced in cryoSPARC v4.2.1 (*e*) Dimer interface schematic with portal (blue) and adaptor (red) models fit within the map using the UCSF ChimeraX Fit Model to Map function. Map density was resolved using D12 symmetry. (*f*) Based on *e*, the dimer interface could be managed by reciprocal salt bridges. (*g*) Two hypotheses can be proposed for the formation of the viral dimer products. Hypothesis A proposes that the ejectosome, DNA and tail assembly are lost after injection and the empty capsids dimerize into the empty-empty or empty-full products. Hypothesis B proposes the portal assembly (*1*), either with or without an ejectosome assembly associated to the portal crown, nucleates the formation of the viral procapsid, subsequently producing two capsid products (ejectosome-full and ejectosome-free). In the ejectosome-free pathway (*top*), the procapsid associates with an adaptor (*2*), at which point DNA packaging is no longer possible as the terminase cannot contact the portal assembly (*3*). The ejectosome-free procapsids dimerize with each other or with a full procapsid at the adaptor-adaptor interface. In the ejectosome-full pathway (*bottom*), the terminase associates with the portal and packages the viral genomic DNA in an ATP-dependent manner (*4*). A dissociation signal is recognized by the terminase which causes the terminase to dissociate from the portal, and an adaptor then associates (*5*). The procapsid can then dimerize with an empty or full procapsid (to form an empty-full or full-full dimer) or can associate with the rest of the tail assembly to form a mature virion (*6*).

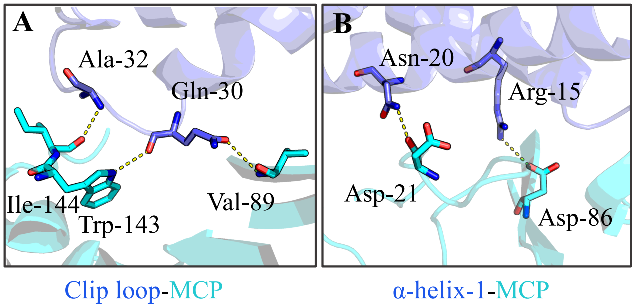

Fig. S2. ΦM1 α-paw. Reciprocal interface interactions which occur between the clip loop (a) and α-helix-1 of the α-paw and the major capsid protein.
